# Supplementary material for: Insights into DNA repeat expansions among 900,000 biobank participants
Source: Nature. 2026 Jan 7;650(8103):920–9. doi: 10.1038/s41586-025-09886-z (PMC12935551; doi:10.1038/s41586-025-09886-z)
Supplement: Supplementary file 1 — Supplementary Methods, Supplementary Figs. 1–4 and Supplementary Tables 1–9. [file 41586_2025_9886_MOESM1_ESM.pdf]

nature portfolio

https://doi.org/10.1038/s41586-025-09886-z

Supplementary information

Insights into DNA repeat expansions among 900,000 biobank participants

In the format provided by the authors and unedited

Methods and supplementary material for "Insights into DNA repeat expansions among 900,000 biobank participants"

Margaux LA Hujoel, Robert E Handsaker, David Tang, Nolan Kamitaki, Ronen E Mukamel, Simone Rubinacci, Pier F Palamara, Steven A McCarroll, Po-Ru Loh

Contents

1 UK Biobank and *All of Us* data sets **3**
1.1 Ethics 3
1.2 UK Biobank data set 3
1.3 *All of Us* data set 3

2 Expanded CAG repeats in UK Biobank **4**
2.1 Extracting WGS reads derived from long CAG repeats 4
2.2 Assigning in-repeat reads to CAG repeat loci of origin 5
2.3 Quantifying enrichment of repeat expansions in transcribed regions 6
2.4 Selection of CAG repeat loci for downstream analysis 6

3 Intergenerational instability of CAG repeats **6**
3.1 Overview of approach and validation 6
3.2 Identifying short and mid-length alleles from spanning reads 7
3.3 Estimating germline mutation rates of short alleles 8
3.4 Validating germline mutation rate estimates 11
3.5 Estimating germline mutation rates of mid-length *GLS* alleles 12

4 Somatic instability of CAG repeats **13**
4.1 Quantifying somatic instability of short CAG repeat alleles 13
4.1.1 Filtering aberrant spanning reads arising from PCR stutter errors 13
4.1.2 Estimating fractions of blood cells harboring somatic mutations 15
4.1.3 Identifying CAG repeat loci with evidence of somatic expansion 16
4.1.4 Assessing efficacy of stringent filtering to deplete PCR stutter errors 17
4.1.5 Replicating age-associated somatic instability of CAG repeats in AoU (v7) 18
4.2 Quantifying somatic instability of long CAG repeat alleles at *TCF4* 18
4.2.1 Estimating lengths of long *TCF4* repeats 18
4.2.2 Estimating relative lengths of inherited *TCF4* repeat alleles 19
4.3 GWAS on somatic expansion of long *TCF4* repeat alleles 21
4.3.1 Optimizing a *TCF4* somatic-expansion phenotype for GWAS 21

4.3.2 GWAS sample inclusion criteria 24
4.3.3 GWAS meta-analysis of somatic repeat expansion in *TCF4* 25

5 Somatic repeat instability beyond CAG repeats 26
5.1 Extracting WGS reads derived from long STRs 26
5.2 Identifying IRRs anchored to their repeat loci of origin 27
5.2.1 Assessing the effect of restricting to known polymorphic STR loci 28
5.3 Determining repeat loci with evidence of somatic mutability 29
5.3.1 Pilot analyses motivating using association with *MSH2* and *MSH3* haplotypes to find unstable STRs 31
5.4 GWAS on somatic expansion of long repeat alleles 31
5.5 Local heritability at DNA repair genes 32
5.6 Quantifying somatic expansion of mid-length AAAG repeat alleles 32
5.6.1 Identifying reads spanning AAAG repeats 32
5.6.2 Estimating mean somatic expansion of an individual's mid-length allele . . 33
5.6.3 Differences in approach compared to spanning-read analyses of CAG repeats 34
5.6.4 Complexities of assessing instability of mid-length alleles of other STRs . 35
5.7 Refined GWAS on somatic expansion of AAAG repeats 36
5.7.1 Optimizing somatic-expansion phenotypes of AAAG repeats for GWAS . . 36
5.7.2 GWAS of AAAG somatic-expansion phenotypes in UKB and AoU (v8) . . 37
5.8 Fine-mapping GWAS signals and computing polygenic scores 38
5.9 Estimating relative expansion rates of groups of individuals 39
5.10 GWAS meta-analysis for mid-length AAAG repeat expansion 40
5.11 Comparison of genetic modifier effects across repeat loci and HD phenotypes . . . 40

6 Phenotypic associations of long repeats 41
6.1 Associations with quantitative traits 42
6.2 Associations with binary disease outcomes 42
6.3 Detailed analysis of the *GLS* locus 43
6.4 Replication of *GLS* associations with liver and kidney disease in AoU (v7) 45
6.5 Assessment of X chromosome repeats for association with phenotypes 45

7 Supplementary Figures 47

8 Supplementary Tables 51

References 60

1 UK Biobank and *All of Us* data sets

1.1 Ethics

This research complies with all relevant ethical regulations. The study protocol was determined to be not human subjects research by the Broad Institute Office of Research Subject Protection and the Partners HealthCare Human Research Committee (as all data analyzed were previously collected and de-identified).

1.2 UK Biobank data set

UK Biobank is a prospective cohort study of half a million volunteer participants aged between 40 and 69 years at recruitment between 2006–2010 [1]. Whole genome sequencing (WGS) data was performed on blood-derived DNA for 490,640 UKB participants [2]. Blood-derived DNA was sequenced using Illumina NovaSeq 6000 sequencing machines to an average coverage of 32.5x, after which 151bp paired-end reads were aligned to GRCh38 and SNP and indel calling was performed as previously described [2].

Blood samples used for WGS had been acquired at initial assessment for 99.6% of sequenced individuals; for the remaining individuals, blood samples from a later visit to a UK Biobank assessment center were used for sequencing. Given the small fraction of participants whose sequenced blood samples had been obtained after initial assessment, we included all individuals in our analyses (using age at initial assessment in analyses of age; averaged across the cohort, this underestimated age at blood draw by $\sim$1 week).

1.3 *All of Us* data set

The NIH *All of Us* Research Program is a longitudinal cohort study which began enrolling participants aged 18 and older in May 2018 and plans to eventually enroll at least 1 million individuals from the United States [3]. We used WGS from the AoU v7 and v8 cohorts [4]. We used v7 for analyses of CAG repeats and v8 for subsequent analyses of AAAG repeats. WGS data in the v7 release was all blood-derived; WGS data in v8 was mostly blood-derived, but $\sim$12% of samples were saliva-derived (365,918 blood-derived and 48,899 saliva-derived samples; see Section 5.7.2). Samples were sequenced to an average coverage of 37.9x on Illumina NovaSeq 6000 instruments. Sequencing reads were aligned to GRCh38 with the Illumina DRAGEN pipeline [4].

2 Expanded CAG repeats in UK Biobank

2.1 Extracting WGS reads derived from long CAG repeats

To characterize the landscape of CAG repeat expansions in a generally-healthy population cohort, we searched for evidence of long CAG repeats in short-read, 151bp paired-end WGS data available for 490,416 UK Biobank participants. Specifically, we searched for in-repeat reads (IRRs), which we operationally defined as read sequences containing $\geq$45 distinct occurrences of a CAG repeat unit (i.e., CAG, AGC, GCA, CTG, TGC, or GCT, to identify CAG repeats on either strand of the GRCh38 human reference genome). We chose the threshold of $\geq$45 to allow for base-calling errors while still ensuring that IRRs were nearly, if not completely, comprised of CAG repeats.

To efficiently identify such IRRs, we made use of the observation that conveniently, bwa [5] had aligned most IRRs (regardless of their locus of origin) to a single locus: the CAG repeat in *TCF4*, which is the longest CAG repeat in the GRCh38 reference genome (Supplementary Fig. 1a). More precisely, among 500 randomly selected WGS samples, 91% of all IRRs were aligned to *TCF4*, and among the remaining IRRs, nearly all either originated from *CA10* or contained a moderate amount of non-CAG flanking sequence and were therefore less relevant to our analyses (Supplementary Fig. 1b,c). This read-mapping behavior, which allowed us to extract most IRRs by analyzing only a single slice of each indexed WGS alignment (cram) file (reducing computational cost by $\sim$2,000-fold compared to scanning the entire cram file), was specific to alignments generated using bwa; in alignments generated by DRAGEN [6], IRRs were more frequently (correctly) mapped to the same loci as their mates.

Specifically, for each WGS cram file containing reads aligned to GRCh38 using bwa, we extracted reads aligned to chr18:55585150-55587230 (which contains the *TCF4* CAG repeat and 1kb of flanking sequence on either side). We excluded reads with any of the last four SAM flags set (`0xF00`). Among the subset of reads that aligned to the *TCF4* CAG repeat sequence (chr18:55586150-55586230), we analyzed read sequences to identify IRRs (as defined above). For each IRR, we further analyzed its mate to determine what information the paired reads provided about their originating sequence:

- If the mate aligned to the $\sim$2kb region chr18:55585150-55587230 (such that it had already been extracted):
  - If the mate was also an IRR, then we recorded the read pair as an "IRR pair" (indicative of a highly expanded repeat; Supplementary Fig. 1).
  - Else, if the mate had mapping quality (MAPQ) $>$ 30, then we recorded the read pair as an "anchored IRR" (Supplementary Fig. 1a) originating from the *TCF4* repeat.
- Else, if the mate aligned to a locus other than *TCF4* (i.e., to a different chromosome, before chr18:55580000, or after chr18:55590000), we extracted the mate from the cram file and

examined its mapping quality:

   – If the mate had MAPQ > 30, then we recorded the read pair as an anchored IRR (known to originate approximately from the mate's aligned position).

2.2 Assigning in-repeat reads to CAG repeat loci of origin

Anchored IRRs provide only approximate information about locations of long repeats, as although one member of the read pair is aligned with high mapping quality, the exact distance between this location and the repeat itself is unknown (due to variability in WGS fragment lengths, which are typically several hundred base pairs but occasionally exceed 1kb). To identify the specific CAG repeat loci from which IRRs originated, we applied the following procedure:

1. Assign anchored IRRs to 100kb bins (based on Mb coordinates of aligned mates, rounded to one decimal place). Most 100kb bins did not contain an anchored IRR in any UKB participant; only 2844, 169, 38, and 27 bins contained an anchored IRR in at least 1, 2, 3, and 5 UKB participants, respectively.
2. Refine 100kb bins containing an anchored IRR in $\geq$5 UKB participants to specific CAG repeats previously genotyped in a high-quality STR reference panel [7]. We downloaded both per-locus summary statistics (including coordinates and repeat unit sequence) and population-specific statistics (including allele frequency and heterozygosity) from `https://github.com/gymrek-lab/EnsembleTR`. We then identified the subset of autosomal repeats with a CAG/CTG repeat unit that were polymorphic within the 1000 Genomes EUR population [8], comprising a total of 1,159 autosomal CAG repeats. Finally, for each of the 25 autosomal 100kb bins with an anchored IRR in $\geq$5 UKB participants, we computed the median (across participants) of the mean anchor location per participant, and we checked whether this median position was within 500bp of the start coordinate of a EUR-polymorphic CAG repeat. This procedure assigned 18 of the 100kb bins to specific autosomal CAG repeats (Supplementary Table 1).
3. Drop anchored IRRs with alignment locations not matching identified CAG repeats. For each of the 18 100kb bins that resolved to a CAG repeat in the previous step, we re-examined individuals with anchored IRRs in these 100kb bins to verify that their alignment locations were near the identified CAG repeat. This was nearly always the case; we found only three individuals with discrepant alignment locations (mean anchoring alignment position $>$10kb from the CAG repeat location) and dropped these individuals from the corresponding lists of anchored IRR carriers (one individual each for *CA10*, *LRMDA*, and *ATN1*).

We further assigned IRR pairs to CAG repeat loci from which they were likely to originate. Because these IRR pairs consisted completely or nearly completely of CAG repeat sequence, they

could not be confidently aligned to loci of origin based on their sequence composition. However, any locus containing a highly expanded repeat generating an IRR pair would be expected to also generate several anchored IRRs (Supplementary Fig. 1). Because most individuals had at most one locus generating many anchored IRRs, we could therefore assign IRR pairs to loci of origin with high confidence by examining counts of anchored IRRs aligned to 100kb bins.

Specifically, if all of an individual's anchored IRRs aligned to the same 100kb bin, we assigned that individual's IRR pairs to this bin. If an individual had anchored IRRs aligned to multiple bins, we assigned IRR pairs to the bin containing the largest number of anchors, excluding the *CA10* locus (because although long *CA10* alleles are common, highly expanded *CA10* alleles are rare: among individuals with anchors only at *CA10*, only ∼1% (1,069 of 122,238) had IRR pairs). In the rare case of a tie between multiple 100kb bins (other than *CA10*) having the same largest number of anchors, we randomly assigned IRR pairs to tied bins.

2.3 Quantifying enrichment of repeat expansions in transcribed regions

Most of the 18 CAG repeat loci for which we detected long alleles in ≥5 UKB participants were in exons. To test whether repeat expansions were enriched in various transcriptional contexts, we intersected the list of 1,159 EUR-polymorphic autosomal CAG repeats with GENCODE v44 canonical transcript annotations to determine the transcriptional context of repeats. We then used Fisher's exact test to assess whether CAG repeats in a given context were more or less likely to be expanded to ≥45 repeat units in ≥5 UKB participants (Supplementary Table 2).

2.4 Selection of CAG repeat loci for downstream analysis

Among the 18 CAG repeat loci identified by the above analyses (Supplementary Table 1), we took forward a subset of 15 CAG repeats for further analysis of germline and somatic mutability based on satisfying the following criteria: (i) heterozygosity >0.01 in EUR [7], (ii) >5 individuals with anchored IRRs in the repeat's 100kb bin, and (iii) only one CAG repeat segment contained within the repeat structure (for ease of genotyping).

3 Intergenerational instability of CAG repeats

3.1 Overview of approach and validation

For each repeat, among individuals whose two alleles differed in length and could be confidently phased to SNP haplotypes, we identified each haplotype's longest IBD partner (usually sharing >10 cM of IBD [9]). For each such IBD pair, we estimated the time to their most recent common ancestor (TMRCA), and we determined the allele carried by the common ancestor by examining

alleles carried on "outgroup" haplotypes (Fig. 1a). Restricting to IBD pairs for which the ancestral allele was confidently determined, we obtained a data set containing hundreds of thousands of ancestral alleles together with the alleles transmitted to pairs of UKB participants (typically descended 10–30 generations; Supplementary Fig. 2a). The large number of allele transmissions represented in this data set—comprising millions of meioses—allowed us to precisely estimate allele-specific, expansion- or contraction-specific mutation rates.

To validate this approach, we verified that estimated intergenerational mutation rates were consistent with rates of discordances between genotypes of sibling pairs (to validate the population-average mutation rate of each CAG repeat locus, i.e., the probability that a randomly-sampled allele mutates in one generation; Supplementary Fig. 2b), and we also verified that probabilities of mutation between ancestral and transmitted alleles scaled linearly with estimated TMRCA (Supplementary Fig. 2a). We further verified that the distribution of mutational jump sizes obtained from this analysis was broadly consistent with distributions previously observed in analyses of de novo mutations [10, 11]: across all 15 repeat loci, 61% of mutations modified CAG-repeat lengths by 1 repeat unit, with 44% being single-repeat-unit expansions and 17% being single-repeat-unit contractions.

Details of the above analyses are provided in the following sections.

3.2 Identifying short and mid-length alleles from spanning reads

For each of the 15 selected CAG repeat loci, we identified *short and mid-length* alleles (<45 repeat units) supported by spanning reads. We used the following pipeline to identify alleles observed within each UKB participant, recording both alele lengths and intra-repeat sequence variations:

1. Extract reads aligned to the repeat locus with mapping quality ≥30 and with mate mapped to the same chromosome.
2. Restrict to spanning reads containing exactly one occurrence each of a starting and ending flank sequence (9bp, consisting of one 3bp repeat unit along with 6 flanking base pairs directly before and after the repeat in the GRCh38 reference genome; Supplementary Table 1).
3. For each allele length supported by a spanning read, determine a consensus sequence for this allele (in the individual being analyzed). Specifically, assign each base of the consensus repeat sequence to be the most commonly-observed base at that position among all spanning reads supporting the repeat length under consideration, restricting to base calls with quality ≥25 (i.e., QUAL of `F` or `:` under the base-quality discretization used in UKB cram files). Set bases with no high-quality base calls or with a tie for the most common base to missing.

To validate that this pipeline robustly genotyped repeat interruptions, we analyzed IBD2 sibling concordance for the common 18-repeat allele at *TCF4* and 17-repeat allele at *CA10* and found that among pairs for whom at least one of the siblings was genotyped as having an interruption (922

for *TCF4* and 336 for *CA10*), the other sibling was also genotyped as having an interruption 100% of the time.

To generate the allele frequency histograms shown in Extended Data Fig. 1b, we computed allele frequencies among individuals we could confidently genotype as heterozygotes (based on observing two distinct alleles with ≥5 spanning reads and no other alleles with >2 spanning reads) or were likely to be homozygotes (based on observing a single allele with ≥10 spanning reads and no other alleles with >2 spanning reads).

3.3 Estimating germline mutation rates of short alleles

We estimated repeat-specific, allele-specific intergenerational expansion and contraction rates by analyzing length discordances among alleles belonging to genomic tracts inherited identical-by-descent (IBD), building upon IBD-based analyses of single-nucleotide mutations [12–16]. We restricted these analyses to common, shorter alleles (≤30 repeat units) that were typically spanned by several short reads, such that alleles were unlikely to "drop out" due to not generating any spanning reads by chance.

At a high level, we implemented this approach by phasing each individual's repeat alleles based on SNP haplotypes, and then for pairs of individuals sharing long extended SNP-haplotypes (i.e., long IBD pairs), we determined the ancestral repeat allele length using less-recent IBD (Fig. 1a) to five "outgroup" haplotypes. Counting repeat-length discordances between long IBD pairs then allowed us to estimate mutation rates, with the ancestral allele being used to determine if the mutation was an expansion or contraction.

In more detail, for each of the 15 CAG repeat loci, we performed analyses within a (locus-specific) subset of individuals heterozygous for short alleles that could be phased with high confidence. That is, we required that individuals have:

- Two distinct alleles each supported by ≥5 spanning reads and no other alleles with >2 spanning reads.
- Length difference of ≥3 repeat units separating the two alleles (i.e., $|A_1 - A_2| \geq 3$), faciliating phasing of the alleles onto the individual's two haplotypes (based on which allele had SNP-haplotype matches with longer repeats; see below). For most loci, the number of available heterozygous individuals did not greatly change whether the threshold was set at ≥1 unit (no filter), ≥3 units, or ≥5 units (Supplementary Table 3); we therefore chose ≥3 units to guard against phasing errors while retaining good statistical power.

We further restricted analyses to individuals with SNP-array genotypes [1] for whom we had previously generated phased haplotypes and identified longest IBD matches [9]. Among these individuals, we performed the following sequence of analyses:

1. **Phase repeat alleles onto SNP-haplotypes** (using IBD matches to determine which of an individual's two haplotypes contains the longer allele). We used an iterative algorithm starting by randomly assigning phase and then running 50 phase-update iterations: for each individual in turn, we computed the mean allele length across the longest five IBD matches for haplotype 1 (respectively, haplotype 2) and then re-assigned phase according to which haplotype was estimated to carry a longer allele.
2. **Identify recent IBD pairs and the ancestral allele of each pair.** For each haplotype of each individual, we examined the haplotype's longest IBD match. If the IBD length was $>$5 cM and spanned $>$0.5 cM on each flank of the CAG repeat locus, we attempted to identify the ancestral allele of the IBD pair using "outgroup" haplotypes sharing less-recent IBD (Fig. 1a):
    - For each of the two haplotypes in the IBD pair, identify its longest 20 IBD matches. Define the set of outgroup haplotypes to be the intersection of these two top-20 sets.
    - If $\geq$5 outgroup haplotypes are found, examine the repeat alleles carried by the top five outgroup haplotypes. If a majority of these alleles ($\geq$3 of 5) match one another (in allele length), and if this consensus allele also matches one or both of the alleles in the IBD pair, designate this consensus allele to be the ancestral allele of the IBD pair. The primary purpose of requiring the consensus allele to match one or both of the alleles in the IBD pair was to eliminate IBD pairs in which the two alleles in the IBD pair match one another but they do not match the predicted allele of the ancestor (imputed from outgroup haplotypes). The most likely reason for this scenario is that a single mutation occurred on a branch of the tree between the ancestor and the outgroup haplotypes (rather than two mutations having occurred, one on each of the two branches connecting the ancestor to the IBD pair). Since we only wished to count mutations along the branches connecting the ancestor to the IBD pair, this scenario needed to be filtered.
3. **Determine an approximately independent set of inferred allele transmissions** (from ancestral alleles to IBD pairs). For each IBD pair with an ancestral allele identified in the previous step, we computed the allele length difference (Δ) between each present-day allele (in the IBD pair) and the ancestral allele. One or both of the alleles was required to have Δ=0 in the previous step. We further required that $\Delta \in \{-2, -1, 0, 1, 2\}$ (reasoning that larger jump sizes might be more likely to arise from genotyping or phasing error). Finally, we removed any duplicate IBD pairs: we had ascertained IBD pairs by examining each haplotype's longest IBD match, such that closely-related haplotypes often nominated each other. This final set of IBD pairs and their inferred ancestral alleles represented an approximately non-redundant set of allele transmissions, as longest IBD matches should typically correspond to leaf-pairs on a coalescent tree.

4. **Estimate the number of generations between each ancestral allele and IBD pair.** To do so, we computed the expected time to the most recent common ancestor (TMRCA) of each IBD pair based on the length of the shared IBD tract and a demographic model accounting for recent population growth.

   If two alleles have a known TMRCA, the distribution of the length (in Morgans) of the IBD segment containing them is given by a gamma distribution with shape parameter 2 and rate parameter $2t$ per Morgan ($f(L|\text{TMRCA} = t) = L(2t)^2 e^{-2tL}$; pages 57–58 of [17]). To compute expected TMRCA given the length of an IBD tract, we can use Bayes' theorem to obtain $P(t|L) \propto f(L|t)P(\text{TMRCA} = t)$. Thus, if L is the length of an IBD segment in Morgans and TMRCA $= t$,

$$\mathbb{E}[t|\text{IBD length} = L] = \frac{\int t^3 e^{-2tL} P(\text{TMRCA} = t)dt}{\int t^2 e^{-2tL} P(\text{TMRCA} = t)dt}. \quad (1)$$

   The term $P(\text{TMRCA} = t)$, which represents a prior on the TMRCA of two alleles, is dependent on demographic history. This probability distribution can be numerically computed assuming that the effective population size $N_e(t)$ at each generation t in the past is known: under a coalescent model, coalescence occurs with probability $1/(2N_e(t))$ at generation t (assuming it has not yet occurred), allowing iterative computation of $P(\text{TMRCA} = t)$ for $t = 1, 2, 3, \ldots$. We used autosomal $N_e(t)$ values that had previously been estimated for the past 300 generations based on an analysis of 10,000 White British UKB participants [18].
5. **Estimate allele-specific per-generation expansion and contraction rates.** For a given allele A, the estimated rate of intergenerational mutational jumps of size c is $\frac{\sum(\Delta==c)}{\sum \text{TMRCA}}$, where the sums are across transmissions within IBD pairs with ancestral allele A. This calculation assumes that at most one mutation occurred between each present-day and ancestral allele, which is a reasonable assumption here (for IBD >5 cM and mutation rates <0.01 per generation). This may lead to mutation rate estimates that are slightly conservative because of the possibility of multiple mutations that would either be counted as a single mutation or filtered from analysis. However, the underestimation is expected to be slight given that the mutation rates that we estimated did not exceed 0.005 for most repeat loci and were all <0.01 (Extended Data Fig. 1b), such that the likelihood of multiple mutations with the typical TMRCA of ~10–30 generations is low (Supplementary Fig. 2).
6. **Estimate confidence intervals of mutation rates.** To do so, we rounded the total number of generations ($\sum \text{TMRCA}$) to the nearest integer and computed a binomial confidence interval (as most haplotypes were used only once; Supplementary Table 3).
7. **Recalibrate mutation rates and CIs to adjust for ascertainment of longest IBD matches.** The procedure above slightly underestimated mutation rates because the above computation of $\mathbb{E}[\text{TMRCA}|\text{IBD length} = L]$ did not account for our ascertainment of only the longest

IBD match for each haplotype. (Intuitively, knowing that an IBD match of length L was the longest IBD match for a given haplotype means that its expected TMRCA is more recent than that of a random IBD match of length L.) To correct for this issue, we computed an approximate calibration factor estimating the extent to which we had overestimated TMRCAs. To do so, we compared mutation rates computed using the above procedure to corresponding computations in which we relaxed the requirement that each IBD pair be the longest IBD match for a haplotype, instead considering all IBD pairs with length >5 cM spanning >0.5 cM on each flank of the CAG repeat locus. (This larger set of IBD pairs contains some redundancy of allele transmissions, making confidence intervals difficult to estimate, but removes ascertainment bias.) Across the 15 CAG repeat loci, we observed that we had underestimated mutation rates by a median factor of 1.204548 (computed by comparing mean rates of $\Delta = \pm 1$ mutations, aggregated across ancestral alleles). We therefore multiplied all mutation rate estimates and confidence interval sizes by this correction factor.

3.4 Validating germline mutation rate estimates

We validated our approach to estimating germline mutation rates using two strategies. First, we examined the empirical probability of observing a length discordance between the two alleles in an IBD pair as a function of their expected TMRCA. If such discordances primarily reflect mutations (rather than genotyping errors or errors in determining IBD), and if expected TMRCA is correctly estimated, then the probability of discordance should scale approximately linearly with TMRCA (e.g., the probability of a mutation occurring among 20 allele transmissions should be roughly twice that of a mutation among 10 allele transmissions). In contrast, error modes that generate either false-positive discordances or noise in estimated TMRCA should flatten this relationship and produce a nonzero intercept at TMRCA=0. We implemented this analysis on the set of IBD pairs used in our germline mutation rate analyses and computed, for each decile of expected TMRCA (adjusted for ascertainment), the fraction of IBD pairs for exhibiting a length discordance of 1 repeat unit (as our analyses focused on 1-repeat-unit expansions and contractions). Across all 15 CAG repeat loci, we observed that discordance probabilities did indeed scale approximately linearly with TMRCA, as expected (Supplementary Fig. 2a).

Second, we directly validated germline mutation rate estimates using IBD2 siblings (i.e., siblings who inherited the same maternal haplotype and the same paternal haplotype at a given locus). The UKB cohort contained ~5,000 IBD2 sib pairs per locus, and while this sample size was insufficient to validate allele-specific mutation rates, it was large enough to provide a check on the population-average mutation rate of each CAG repeat locus (i.e., the probability that a randomly-sampled allele mutates in one generation). We first computed this value using all IBD pairs used in our germline mutation rate analyses (by dividing the total number of 1-repeat-unit discordances by twice the total TMRCA, adjusted for ascertainment). We then computed the corresponding value

within IBD2 sibs, applying an analogous set of filters to the individuals included in analysis (i.e., restricting to confidently-phased heterozygotes and restricting to allele pairs that differed in length by $\Delta \in \{-2, -1, 0, 1, 2\}$) and then computing an analogous population-average mutation rate (by dividing the total number of 1-repeat-unit discordances by twice the number of allele pairs, since each allele pair had TMRCA=1). We computed a confidence interval for the IBD2-sib-based estimate using an exact binomial test as implemented in `binom.test` in `R` [19] and observed that for all 15 CAG repeat loci, the IBD2-sib-based confidence interval contained the estimate based on our full analysis, and the point estimates from the two approaches were broadly consistent (Supplementary Fig. 2b).

3.5 Estimating germline mutation rates of mid-length *GLS* alleles

Common, short alleles of the *GLS* 5' UTR repeat ($\leq$24 repeat units) exhibited particularly high germline mutation rates relative to the other CAG repeats we analyzed, leading us to wonder how much more quickly rare, mid-length *GLS* alleles (25–40 repeat units) might mutate. Assessing the mutation rates of these longer alleles using the analysis pipeline described above was not feasible because of the rarity of these alleles and the smaller numbers of spanning reads typically available for genotyping them (due to their longer lengths). Instead, we studied the mutation rates of these alleles using a separate pipeline, analyzing allele length discordances among close relatives in UKB based on DRAGEN genotyping [6] (which more optimally utilized available read data). UK Biobank provided DRAGEN genotype calls at 39 STR loci, one of which was *GLS*, so we used the DRAGEN genotypes for this analysis.

We first identified IBD pairs with mid-length alleles using the following procedure. For each individual that DRAGEN had genotyped to carry one allele of length $\geq$25 repeat units (supported by $\geq$1 spanning read) and another allele of length $<$20 repeat units, we identified the five longest IBD matches of each of the individual's two haplotypes. We phased the individual's short allele onto the haplotype for which the larger fraction of individuals sharing longest IBD with that haplotype carried the short allele. The mid-length allele was thus phased onto the opposite haplotype, such that the top IBD matches of that haplotype constituted IBD pairs of interest. For each such IBD pair, we assumed that the other individual's longer allele was the shared allele (as the large majority of *GLS* alleles are short; mid-length and long *GLS* alleles are rare).

We then analyzed the subset of these IBD pairs that involved close relatives (third-degree or closer based on previously-computed kinship coefficients ϕ [1]). To estimate germline mutation rates, we divided the number of discordances among IBD pairs by the sum of the numbers of generations separating these pairs, estimated as $-\log_2(\phi)$ (reasoning that full-sib, avuncular, and first-cousin relationships were likely to comprise most such relationships). We estimated germline mutation rates separately for haplotype-pairs for whom the shortest mid-length allele among the pair had length 25–29 repeats or 30–34 repeats. There were 214 pairs of close relatives (428

individuals; 390 unique individuals) for whom the shortest mid-length allele among the pair had length 25–29 repeats or 30–34 repeats. As above, we estimated confidence intervals by rounding the total number of generations to the nearest integer and computing a binomial confidence interval, which was a reasonable approximation given that only a small fraction of individuals appeared in multiple pairs of closely related haplotypes.

4 Somatic instability of CAG repeats

4.1 Quantifying somatic instability of short CAG repeat alleles

An initial analysis of common, short alleles of the *GLS* repeat ($\leq$24 repeat units) also showed clear evidence of cases of somatic mosaicism: for 15 UKB participants, WGS data indicated the presence of three distinct alleles (each supported by $\geq$5 spanning reads), and these individuals were $\sim$5 years older than average (mean age of 61.3 years). This observation led us to try to quantify somatic instability of alleles short enough ($\leq$30 repeat units) to typically be spanned by several WGS reads.

4.1.1 Filtering aberrant spanning reads arising from PCR stutter errors

We hypothesized that we might be able to find evidence of somatic mutation from single spanning reads suggesting a $\pm$1 or $\pm$2 repeat unit mutation relative to an inherited allele (supported by many spanning reads). The main challenge of such an analysis is that such aberrant reads commonly arise from "PCR stutter" error caused by polymerase slippage during PCR amplification [20–22]. This error mode arises even in WGS data generated from libraries prepared using PCR-free protocols (which were used by UKB and AoU) because of the "bridge amplification" step of sequencing by synthesis, in which a DNA fragment to be sequenced is PCR-amplified into a localized cluster of (usually identical) single-stranded DNA molecules anchored to a flow cell [23] (Fig. 1c). Sequencing is then performed by iteratively adding fluorescently tagged nucleotides complementary to these single-stranded DNA molecules (beginning with a primer and extending the complementary DNA strand one base pair at a time) and measuring the fluorescence signal generated (in aggregate) by the nascent nucleotide of each molecule in the cluster. At each successive iteration, a base call (corresponding to the nucleotide position just added) is derived from this fluorescence signal.

When bridge amplification occurs without error, the DNA molecules that form each clonal cluster are identical, such that at each iteration of sequencing by synthesis, the same new nucleotide is added to the complementary DNA strand of each molecule in the cluster, and the same fluorescence signal is emitted from each newly-added nucleotide in the cluster, generating a high-quality base call. However, if a PCR stutter error occurs duing bridge amplification, a polyclonal cluster

containing a mixture of molecules (some containing the error and others not containing the error) is produced (Fig. 1c). Consequently, at some base positions, molecules with and without the error will contain different nucleotides, such that the aggregate fluorescence signal generated by the polyclonal cluster will contain a mixture of signals corresponding to the distinct nucleotides represented in the cluster. In this scenario, the base call that is made might correspond to either the original sequence or the error-modified sequence, and—importantly for our purposes—the base quality (i.e., base call confidence) will be reduced.

The upshot of the above behavior is that when an aberrant spanning read (supporting a repeat allele that appears to harbor a length mutation) is generated by a PCR stutter error during bridge amplification, the base quality string corresponding to this read sequence should contain a *predictable* sequence of low-quality bases: read positions through the end of the repeat should have high quality (because the two species of DNA molecules present agree up to this point), after which positions at which the molecules with and without the error have inconsistent nucleotides should have low quality (Fig. 1c). Examining base qualities of aberrant spanning reads (some of which reflect real somatic mutations, but many of which arise from PCR stutter errors) showed that for many reads, this pattern was indeed readily visible (Fig. 1c and Supplementary Fig. 3a).

Based on these observations, we devised a filtering strategy to identify a high-quality set of aberrant spanning reads in which real somatic mutations were enriched and PCR stutter errors were depleted. The main idea of this filtering strategy was to identify reads with high base qualities at the positions at which PCR stutter error would be expected to reduce base qualities. This filter was not expected to be perfect, particularly because polymerase slippage during the initial step of bridge amplification (in which the original DNA fragment to be sequenced hybridizes to an oligo on the flow cell, a polymerase creates the complement of the hybridized fragment, and the original fragment washes away) produces a monoclonal cluster in which all DNA molecules contain the PCR stutter error, leaving no evidence of error in the base quality string. Nonetheless, for repeats with relatively higher somatic mutation rates, and for alleles of longer lengths (which are more prone to somatic mutation), we reasoned that somatic mutations could be sufficiently common to represent the majority of aberrant reads that passed stringent filtering, allowing analysis of somatic mutation of short repeat alleles from UKB and AoU WGS data.

Specifically, we applied the following analysis pipeline to each CAG repeat locus:

- Restrict to heterozygous individuals with exactly two alleles supported by $\geq$3 spanning reads, with these two alleles differing in length by $\geq$5 repeat units ($|A_1 - A_2| \geq 5$). In such individuals, we could confidently determine which allele a putative mutation (supported by an aberrant spanning read) arose from.
- Identify aberrant alleles differing in length from an inherited allele by ±1 or ±2 repeat units and supported by exactly one aberrant spanning read.
- Filter aberrant spanning reads to those with high base qualities at diagnostic positions and

with robustly-determined allele lengths. For each aberrant spanning read, we assumed that the aberrant allele originated (either due to somatic mutation or PCR stutter error) from the inherited allele closer in length. We then determined which diagnostic read positions would be expected to have low quality had the aberrant allele been produced during bridge amplification. To do so, we shifted the sequence of the appropriate flank—right flank for reads aligned in forward orientation; left flank for reads aligned in reverse orientation—by $\pm$3bp or $\pm$6bp toward the repeat (corresponding to the other sequence that would be expected to contribute to the cluster, had a PCR stutter error occurred) and identified positions at which the unshifted sequence differed from the shifted sequence (Fig. 1c). Finally, we required that the aberrant spanning read satisfy all of the following filters:

- $\geq$4 diagnostic base positions identified.
- Maximal base quality (`F`, i.e., Q score of 37) observed at $\geq$80% of diagnostic positions.
- Perfect or near-perfect match to the expected sequence on the opposite flank (left flank for forward reads; right flank for reverse reads). This filter ensured that base positions sequenced before the repeat sequence were accurately sequenced and were consistent with the read truly being a spanning read. Specifically, we required $\geq$95% sequence identity (compared to the consensus sequence of the originating inherited allele) among high-quality bases (Q$\geq$25; i.e., QUAL of `F` or `:`) on the first-to-be-sequenced flank.
- High base quality (`F` or `:`) at any key bases at which a single miscalled base would result in incorrect allele sizing. For example, the left flank of the *TCF4* repeat contains the sequence AGGAG**G**AG**C**AGC; the two bolded bases are key bases because a G-to-C base-calling error in the first base would result in an additional AGC repeat unit, and a C-to-G error in the second base would result in one fewer AGC repeat unit.

4.1.2 Estimating fractions of blood cells harboring somatic mutations

The stringent filtering procedure described above identified a set of aberrant spanning reads free of detectable evidence of PCR stutter error and thus representing putative somatic mutations. However, to use these reads to quantify the propensities of different alleles to mutate somatically (e.g., the rate at which an allele of length L repeats mutates somatically to an allele of length $L+1$), we needed to compute a normalization factor. Specifically, we needed to know how many reads we would expect to pass the same stringent filters in a heterozygous individual who inherited an L-repeat allele but in whom this allele had mutated to length $L+1$ in 100% of blood cells. Given this latter quantity, we could then estimate the fraction of blood cells in which an $L \to (L+1)$ mutation had occurred (averaged across biobank participants who inherited a single L-repeat allele) by dividing the mean number of aberrant spanning reads supporting an $L \to (L+1)$ mutation (and passing filtration) by this denominator.

To estimate the above denominator, we identified individuals who were heterozygous for a

presumably-inherited allele of length $L+1$ and then filtered spanning reads supporting the $(L+1)$-repeat allele as if we were evaluating them for having arisen from somatic mutation or PCR stutter error modifying an L-repeat allele. The average number of $(L+1)$-repeat-spanning reads that passed filtration (per heterozygous carrier of an $(L+1)$-repeat allele) gave an estimate of the desired denominator.

Putting this all together, we estimated the fraction of blood cells harboring an $L \to (L+1)$ mutation as:

$$\frac{\text{Mean number of aberrant reads supporting an } L \to (L+1) \text{ mutation and passing filtration}}{\text{Mean number of reads spanning an } (L+1)\text{-repeat inherited allele and passing filtration}} \quad (2)$$

where the mean in the numerator is across the subset of individuals heterozygous for an L-repeat allele described above, and the mean in the denominator is across individuals heterozygous for an $(L+1)$-repeat allele.

The same approach was also applicable for estimating fractions of blood cells in which L-repeat alleles had mutated to alleles of length $L-1$ or $L \pm 2$, but only somatic mutations to length $L+1$ appeared to occur sufficiently frequently relative to PCR error to obtain robust results.

4.1.3 Identifying CAG repeat loci with evidence of somatic expansion

To assess which of the 15 CAG repeat loci we analyzed showed evidence of somatic instability of common, shorter alleles, we examined the fraction of cells (among UKB participants heterozygous for a given allele) estimated to harbor a 1-unit expansion (respectively, 1-unit contraction) of each common allele of length $\leq$30 repeats (Extended Data Fig. 3). For most CAG repeat loci, estimated fractions of cells with putative contractions exceeded those with putative expansions, suggesting that residual PCR stutter error (which is contraction-biased [20]) could be dominating these estimates. However, for four loci (*GLS*, *ATN1*, *TCF4*, and *DMPK*), putative expansions began to outnumber putative contractions for less-short alleles, typically beyond an allele length of $\sim$15 repeats.

To formally test each CAG repeat locus for evidence of somatic expansion (respectively, contraction), we tested whether the age of a UKB participant associated with whether or not an individual's WGS data contained an aberrant spanning read that passed filtration and supported a 1-repeat-unit expansion (respectively, contraction). We restricted this analysis to UKB participants with at least one allele of length $\geq$15 and included the length of each individual's longer allele as a covariate.

Three loci exhibited Bonferroni-significant associations ($p < 0.05/15$) between age and the presence of a sequencing read putatively derived from a 1-repeat-unit somatic expansion (*GLS*, *TCF4*, and *DMPK*). Additionally, we noticed that *ATN1* apppeared to exhibit a strong increase in estimated somatic expansion rate and an expansion-vs.-contraction skew at slightly longer allele

lengths (Extended Data Fig. 3), so we looked more carefully for an age effect in the precise allele range of potential somatic expansion ($\geq$18 repeats) and observed a significant association with age ($p < 0.05$).

4.1.4 Assessing efficacy of stringent filtering to deplete PCR stutter errors

We performed two analyses to assess the effect our filtering approach. First, for each allele length L at each CAG repeat locus, we evaluated the impact of the filter on our estimate of the fraction of blood cells harboring an $L \rightarrow (L+1)$ mutation. That is, we compared the estimate we obtained using the analytical pipeline described above to an analogous computation in which we did not attempt to filter PCR errors (i.e., counting all spanning reads supporting an $(L+1)$-repeat allele in both the numerator and denominator of equation (2)). As expected, filtering reduced estimated rates of somatic mutation (Supplementary Fig. 3b), indicating that PCR stutter errors were a source of aberrant spanning reads.

Second, we evaluated the efficacy of the filter in enriching for true somatic mutations among aberrant spanning reads. As noted above, we did not expect the filter to completely eliminate PCR stutter error (particularly because errors introduced during the initial step of bridge amplification are undetectable), so we wished to estimate what fraction of aberrant spanning reads that survived filtering represented real somatic mutations (rather than technical artifacts).

To do so, we analyzed the extent to which the estimated fraction of blood cells harboring an $L \rightarrow (L+1)$ mutation increased with age. Assuming that such somatic mutations accrue at an approximately constant rate with age, we would expect the fraction of cells carrying a mutation to scale linearly with age, whereas technical artifacts that produce aberrant spanning reads should be observed at a frequency independent of age. We therefore regressed $y =$ estimated fraction of blood cells harboring an $L \rightarrow (L+1)$ mutation on $x =$ age, including an intercept term in the regression, and we then divided the intercept by the mean y-value to obtain the relative contribution of technical artifacts to the y-values we had measured. Subtracting this quantity from 1 gave an estimate of the effectiveness of our filtering strategy.

To reduce noise in these regressions, we restricted analyses to alleles with >1,000 carriers, and we performed analyses on groups of alleles of lengths $L \leq$10, 11–15, 16–20, 21–25, and $\geq$26 repeats. Additionally, we performed these analyses in the *All of Us* cohort (v7; n=245K WGS) as its age range (18–90+ years) was much wider than UKB (40–70 years), facilitating regression analysis on age. These analyses indicated that for *GLS* and *TCF4*, the majority of aberrant spanning reads that passed filtering were indeed of somatic origin, and the same was true for longer (more mutable) alleles of *ATN1* and *DMPK* (Supplementary Fig. 3c).

4.1.5 Replicating age-associated somatic instability of CAG repeats in AoU (v7)

To evaluate the robustness of our estimates of fractions of blood cells harboring 1-repeat-unit somatic expansions, we compared estimates we obtained by analyzing WGS data from the UKB and AoU (v7) cohorts. We subdivided each cohort into three age tranches and computed estimates within each age tranche for each of the four CAG repeat loci that we had found to exhibit detectable somatic instability in blood (*GLS*, *ATN1*, *TCF4*, and *DMPK*). We observed concordant estimates between the two cohorts, and in both cohorts, somatic expansions were estimated to be more frequent among individuals of older ages (Extended Data Fig. 4).

4.2 Quantifying somatic instability of long CAG repeat alleles at *TCF4*

Some CAG repeat loci are known to be somatically unstable, such that observations of highly expanded alleles ($\sim$100+ repeats, producing IRR pairs) might indicate somatic expansion. To see if we had evidence of somatic expansion in UKB, we computed the average age among carriers of highly expanded alleles of each CAG repeat. In UKB, carriers of highly expanded *TCF4* repeat alleles were significantly older than average (+2.48 years; s.e. 0.08 years), suggesting a contribution of somatic expansion to these particularly long alleles. *TCF4* was the only locus for which carriers of highly expanded alleles were significantly older than average, so we focused further analyses of somatic instability of long repeats on the *TCF4* locus.

4.2.1 Estimating lengths of long *TCF4* repeats

To more directly assess somatic expansion of long *TCF4* repeat alleles in UKB and AoU, we needed a way to estimate lengths of these alleles from short-read WGS data. Counts of in-repeat reads (IRRs) provided a straightforward way to do so, as the number of such reads observed in an individual heterozygous for a long allele ($\geq$45 repeat units) increases approximately linearly with the length of the allele and with autosomal sequencing coverage:

$$\text{Estimated allele length (in repeat units)} = 45 + (\text{\# IRRs}) \times \frac{X}{\text{coverage}} \times \frac{1}{3}, \tag{3}$$

where 45 is the number of repeat units required for an allele to produce IRRs (according to our definition of IRR), $\frac{X}{\text{coverage}}$ is a constant calibration factor estimating that a new read is generated every $\frac{X}{\text{coverage}}$ base pairs of *TCF4* repeat sequence, and $\frac{1}{3}$ converts base pairs to repeat units. For an individual who is mosaic for long alleles of different lengths, this formula estimates the mean length across alleles present in the sequenced DNA sample.

We estimated the calibration factor X by comparing short-read and long-read sequencing data among heterozygous carriers of long *TCF4* alleles for whom long-read data were available in AoU v7. Specifically, we set the median repeat length estimated from long-read data to equal

the median repeat length estimated from short-read data (using the above formula), which gave $X \approx 424$. We used the following procedure to measure *TCF4* repeat lengths from long reads. For each carrier of a long *TCF4* allele (identified from short-read analysis), we identified long reads mapping to the *TCF4* repeat in GRCh38 with mapping quality $\geq$30, excluding reads with any of the last four SAM flags set (`0xF00`). We extracted the segment of each read containing the CAG repeat sequence by searching for exact matches to the left and right flanks of the repeat sequence (AGGAGGAG**CAGCAG** and **CAGCAG**CATGAAA); we verified that this approach generally gave the same result as identifying the first and last occurrences of a string of five CAGs (CAGCAGCAGCAGCAG). We then estimated the length of the repeat allele by computing the number of base pairs between the left and right flanks, dividing by 3, and adding 1. To guard against the possibility of a technical artifact producing extraneous, non-CAG sequence between the flanks, we required that this estimate agree with the number of CAG substrings found within the read sequence to within 50 repeat units.

4.2.2 Estimating relative lengths of inherited *TCF4* repeat alleles

The length of a long *TCF4* repeat allele (estimated from short-read WGS data as described above) reflects both the effect of somatic expansion and the length of the allele that was originally inherited. To increase statistical power to analyze somatic expansion of *TCF4* repeats, we wished to control for inter-individual variation in inherited *TCF4* allele lengths. Doing so directly was not possible given that DNA sequencing data was available from a single time point per individual. Instead, we approximately estimated the relative length of each of an individual's two inherited *TCF4* repeat alleles (relative to the alleles inherited by other sequenced individuals) by using statistical imputation from other individuals who shared an extended—typically multi-megabase—SNP-haplotype at *TCF4* (and were therefore likely to have co-inherited a *TCF4* repeat allele of the same or similar length). Measurements of *TCF4* repeat alleles in these distantly-related individuals were themselves inexact estimates of the lengths of the alleles that these individuals had inherited (both because of sampling noise and because of somatic expansion), but we reasoned that averaging across multiple reference individuals would mitigate the impact of noise, and that while imputed allele lengths overestimate the lengths of longer inherited alleles more so than less-long inherited alleles (due to greater rates of somatic expansion), they still provide a useful measure of the relative lengths of inherited alleles. In detail, the imputation pipeline proceeded as follows:

1. **Computing coverage-adjusted counts of in-repeat reads (IRR).** As noted above, the number of IRRs generated by a *TCF4* repeat allele increases approximately linearly with the length of the allele and with WGS coverage, such that coverage-adjusted IRR count provides a quantification of allele length. In our imputation pipeline, we computed coverage-adjusted IRR count using a slightly different method than described above (simply because we ran

the imputation analysis before working out details of equation (3)):

- In UKB, we computed each sample's coverage-adjusted IRR count (for *TCF4*) as $(\#_{\text{anchored IRRs at } TCF4} + 2 \times \#_{\text{IRR pairs}})/(\text{cov}_{\text{GC67}}/\overline{\text{cov}_{\text{GC67}}})$, where cov_{GC67} denotes a WGS sample's mean autosomal coverage in 400bp windows with 67% GC content (following the GC normalization approach of Genome STRiP [24]), and $\overline{\text{cov}_{\text{GC67}}}$ denotes the mean of this value across UKB WGS samples. We set coverage-adjusted IRR count to 0 (overriding the above formula) if an individual either had no anchored IRRs at *TCF4* or had been genotyped to be heterozygous for two short alleles.
- In AoU, we normalized using the genome-wide coverage metric provided with the AoU v7 WGS data release; i.e., we computed $(\#_{\text{anchored IRRs at } TCF4} + 2 \times \#_{\text{IRR pairs}})/(\text{cov}/\overline{\text{cov}})$ and set this value to 0 if an individual had no anchored IRRs at *TCF4*.

2. **Identifying and phasing carriers of long *TCF4* alleles.** We defined "long-allele carriers" as individuals with nonzero coverage-adjusted IRR count, and in each data set (UKB and AoU), we created an imputation reference panel comprised of the subset of long-allele carriers who had no anchored IRRs at any locus other than *TCF4* or *CA10* (such that IRR pairs could be assumed to nearly always be derived from *TCF4*). We defined SNP-haplotypes of these individuals in the genomic region surrounding *TCF4* using our previous phasing of the UKB SNP-array data set [25] and using `phase_common` from SHAPEIT5 [26] to phase the AoU v7 SNP-array data set. We then determined which haplotype(s) of each long-allele carrier contained a long *TCF4* allele using the following phasing approach:

   - For each of an individual's two haplotypes $i = 1, 2$, we identified the top 20 longest SNP-haplotype matches at *TCF4* in the reference panel (quantified using IBS length as in ref. [27]), and we then computed the fraction f_i of long-allele carriers among the reference individuals in whom these top 20 SNP-haplotype matches were found.
   - If both $f_1 > 0.5$ and $f_2 > 0.5$, we considered the individual to be homozygous-long.
   - Otherwise, we considered the individual to be a heterozygous carrier of a single long allele, and we assigned the long allele to the haplotype i with higher f_i.

3. **Computing an inherited *TCF4* allele length metric by imputing coverage-adjusted IRR count**. For each long-allele carrier, for each haplotype that we determined above to carry a long *TCF4* allele, we imputed coverage-adjusted IRR count from other haplotypes in the reference panel. In UKB, we selected reference haplotypes based on IBD matches that we had previously computed [9], choosing the top 10 longest IBD matches to heterozygous long-allele carriers in the reference panel (and using all such IBD matches if fewer than 10 were available). We then computed the weighted mean of these reference individuals' coverage-adjusted IRR counts (capped at 25), using weights proportional to $\exp(-c/(\text{IBD length}))$ in order to prioritize more recent IBD-sharing. We tuned the c parameter to maximize the

correlation between an individual's own coverage-adjusted IRR and the imputed quantity, selecting $c = 8$ cM in UKB. In AoU, which contained much less IBD-sharing than UKB, we used IBS length instead of IBD and selected $c = 2$ cM.

As noted above, this imputation approach provided imperfect estimates of inherited allele lengths for multiple reasons, including imputation error (arising from germline mutation of the *TCF4* repeat), sampling noise in IRR counts of reference individuals, and upward bias caused by somatic mutation of inherited alleles in reference individuals. However, even rough estimates of relative lengths of inherited *TCF4* alleles were still useful for exploring first-order effects of inherited allele length on somatic mutability (e.g., by stratifying estimated lengths of long *TCF4* alleles (in heterozygotes in the reference panel) by inherited allele length quantile and age; Extended Data Fig. 5a) and allowed us to partially control for these effects in downstream association analyses.

4.3 GWAS on somatic expansion of long *TCF4* repeat alleles

4.3.1 Optimizing a *TCF4* somatic-expansion phenotype for GWAS

To reduce noise in estimates of long *TCF4* allele lengths, we computed an alternative metric based on the number of sequenced DNA fragments derived from a highly expanded repeat. Such fragments, which manifest in WGS data as in-repeat read pairs (IRR pairs; Extended Data Fig. 1a), begin to be observed in 151bp paired-end sequencing data when alleles expand to $\sim$100 repeat units. The specificity of such read pairs to highly expanded alleles thus reduces the effect of sampling noise on estimates of allele lengths in this most-unstable length range (Extended Data Fig. 6a,b). The IRR pair metric associated strongly with age ($p = 1.3 \times 10^{-235}$, controlling for imputed allele length and capping the count at 5; for comparison, IRR-count-based allele length measurements had age associations of $p = 1.5 \times 10^{-116}$ and $p = 6.5 \times 10^{-138}$ before and after controlling for imputed allele length). Among individuals in the top two quintiles of imputed allele length, mean counts of IRR pairs increased several-fold across the age range of UKB participants (Extended Data Fig. 5b).

Quantifying evidence of highly expanded *TCF4* alleles from sequencing data. In light of our observation that counts of IRR pairs in WGS data (indicative of highly expanded alleles; $\sim$100+ repeat units) were particularly age-associated and thus informative of somatic expansion (Extended Data Fig. 5a,b and Extended Data Fig. 6a,b), we used this metric as the "raw data" forming the basis for a *TCF4* somatic-expansion phenotype. To increase statistical power, we also computed a similar metric using whole-exome sequencing (WES) data, which provided an independent quantification of *TCF4* expansion for most UKB participants ($\sim$470,000; ref. [28]). In detail:

- **WGS-derived *TCF4* expansion metric.** Beyond counting IRR pairs, we also assigned a count of 0.5 to individuals with no IRR pairs but with at least one read pair that barely

missed the cutoff for being an IRR pair (i.e., one read in the pair qualified as an IRR based on containing $\geq$45 CAGs, and its mate contained $\geq$40 CAGs). We considered adjusting this metric for WGS coverage and mean fragment length but observed a negligible impact on downstream GWAS power (which was unsurprising given that sampling noise was the primary source of noise in counts of IRR pairs; Extended Data Fig. 6b), so we did not adjust for these WGS parameters.

- **WES-derived *TCF4* expansion metric.** We computed a similar metric from WES data, though some additional care was required because (i) WES reads were much shorter than WGS reads (76bp versus 151bp, resulting in lower mapping qualities and many IRR pairs being flagged as potential duplicates) and (ii) technical variation in WES sequencing depth profiles. To handle these issues, we first identified WES read pairs for which:
  - both reads mapped to the *TCF4* CAG repeat in GRCh38 (either mapping completely within the CAG region or partially overlapping it),
  - at most 10 base pairs of each read were unmapped,
  - one read aligned on the forward strand and the other on the reverse strand, and
  - SAM flags `0xB00` were excluded (but duplicate reads were allowed).

  Among these read pairs, we counted the numbers of:
  - IRR pairs (defined based on both reads mapping completely within the CAG region)
  - "IRR+flank" read pairs (for which (a) the left read was an IRR and the right read spanned the right endpoint of the CAG repeat, or (b) the left read spanned the left endpoint of the CAG repeat and the right read was an IRR).

  We adjusted each of these counts for WES coverage and for duplicate read rate. We then further normalized the adjusted IRR pair count by using the adjusted IRR+flank count to control for WES batch effects (since the adjusted IRR+flank count is expected to be the same for all long-allele carriers, up to technical variation in WES sequencing depth profiles). To do so, we computed an individual-specific correction factor equal to the median adjusted IRR+flank count among long-allele carriers (as assessed using WGS data) represented among 300 individuals with similar WES depth profiles [29] (as well as the individual being normalized). We then computed the WES-derived *TCF4* expansion metric by dividing an individual's adjusted IRR pair count by this correction factor.

Converting *TCF4* expansion metrics and imputed allele length into a somatic-expansion phenotype by predicting age. The *TCF4* expansion metrics we computed from WGS and WES data, together with the rough estimates of relative lengths of inherited alleles that we obtained from imputation, provided the main pieces of information we needed to assess an individual's level of somatic expansion. However, the optimal way in which to combine these measurements

into a GWAS phenotype was not immediately clear. Ideally, we wished to compute an individual's expected genetic liability for somatic expansion based on these observed quantities. However, the precise relationship between IRR pair counts from sequencing data, imputed allele length, and genetic propensity for expansion was presumably a complicated, nonlinear function, such that running GWAS on an IRR pair count metric and including imputed allele length as a covariate would achieve suboptimal power.

We realized that a more-optimal way to utilize these data was to use mean age as a proxy for the extent to which measurements of WGS- or WES-derived *TCF4* expansion and imputed allele length were informative of somatic expansion. The intuition behind this approach is that if people with a given IRR pair count-derived expansion metric and a given imputed allele length tend to skew older (respectively, younger), this tells us that these measurements generally correspond to higher (respectively, lower) levels of somatic expansion.

To implement this approach, we fit a 9-parameter model to predict age from an individual's IRR pair count-derived *TCF4* expansion metric (either WGS-based or WES-based) and imputed allele length (taking the longer of the two imputed lengths in individuals homozygous for a long *TCF4* allele). We parameterized this model to be able to capture the main nonlinearities of this relationship (Extended Data Fig. 6c):

- 2 parameters $z_{\mathrm{min}}, z_{\mathrm{max}}$ determined cropping thresholds for the *TCF4* expansion metric z (e.g., number of IRR pairs) beyond which smaller or larger values of the metric did not provide additional information about somatic expansion
- 3 parameters defined a quadratic function approximating how age varied as a function of imputed allele length among individuals with the smallest value(s) of the *TCF4* expansion metric (specifically, those cropped to z_{min})
- 3 parameters defined a quadratic function approximating how age varied as a function of imputed allele length and age among individuals with the largest values of the *TCF4* expansion metric (specifically, those cropped to z_{max})
- 1 parameter defined an interpolation function determining how to interpolate between the above two curves for individuals with intermediate values of the *TCF4* expansion metric: this function had a value of 1 at z_{min} and 0 at z_{max}, and the single parameter allowed the interpolation weight to vary nonlinearly in between (via a quadratic function of z).

We optimized these 9 parameters using `optim()` in `R` to minimize the mean squared error (MSE) between predicted and actual age. We could then use the fitted model to predict an individual's age (as a proxy for somatic expansion) given their IRR pair count-derived *TCF4* expansion metric (either WGS-based or WES-based) and imputed allele length (longer of two alleles for hom-long individuals).

We fit the model independently for UKB WGS (Extended Data Fig. 6c), UKB WES, and AoU WGS, obtaining qualitatively similar behavior of these three model fits. To combine WGS-based

predicted age and WES-based predicted age into a single somatic-expansion phenotype in UKB, we computed a weighted average of the WGS-based and WES-based predicted ages, selecting the weight that produced the largest correlation with actual age ($\approx$ 71% WES + 29% WGS). For UKB participants with WGS data but no WES data, we used the WGS-based predicted age phenotype alone.

4.3.2 GWAS sample inclusion criteria

To enable robust, well-powered genome-wide association analysis of somatic expansion of *TCF4* repeats in UKB and AoU (v7), we applied the following sample selection filters.

Long-allele carriers. We restricted to individuals who carried long *TCF4* alleles ($\geq$45 repeat units) as described in Section 4.2.2, given that only long alleles were sufficiently unstable to contribute meaningfully to GWAS power. Long-allele carriers represented $\sim$8% of individuals of European genetic ancestry and were a few times less common in non-European ancestries.

Age and relatedness. In UKB, we did not apply any filters on age (which ranged from 40–70 years old at DNA acquisition) or relatedness (because we performed GWAS in UKB using a linear mixed model, which controlled for relatedness). In AoU, which had a wider age range (18–90+ years), we restricted to individuals of age $\geq$40, and we further restricted to a subset of unrelated individuals by removing the younger individual in each pair of relatives with kinship coefficient $>$0.1.

Genetic ancestry. In UKB, we inferred the genetic ancestry of each participant using their coordinates along the top 20 genetic principal components (PCs). We defined EUR, SAS, AFR, and EAS cluster centers by taking mean PC coordinates among individuals with self-reported ethnic group "White," "Asian or Asian British," "Black or Black British," and "Chinese," respectively, given that self-reported ethnic groups in UKB were previously observed to broadly match the genetic ancestries of 1000 Genomes continental population groups [1]. We then assigned individuals to genetic ancestry clusters based on their Euclidean distances (in PC-space) to these cluster centers. Specifically, for each cluster, we examined Euclidean distances from that cluster center among individuals of the corresponding ethnic group, and we defined a cluster radius that included 99% (for EUR) or 90% (for SAS, AFR, and EAS) of these individuals. We then used these PC-based cluster definitions to assign individuals to genetic ancestry clusters (irrespective of their reported ethnic groups), leaving individuals who did not fall within any cluster unassigned.

In AoU, we used genetic ancestry assignments that had previously been computed [4].

To safeguard against confounding from population stratification, we restricted GWAS to individuals of EUR genetic ancestry in UKB and AoU. We also performed a version of the GWAS that

lifted this restriction (instead including genetic ancestry as a covariate) and observed no appreciable difference in the results, which was unsurprising given that the large majority of long-allele carriers in the combined data set (>90%) were of EUR genetic ancestry.

The filters above resulted in GWAS sample sets of size n=40,231 in UKB and n=8,217 in AoU. In addition to these filters, we applied a final sample exclusion criterion that varied based on the genetic variant being tested for association with somatic expansion.

Chromosome-specific exclusions of individuals with long CAG repeats at loci other than *TCF4*. Counts of IRR pairs (on which our *TCF4* somatic-expansion phenotype was based) primarily reflected repeat length variation at *TCF4*, but in individuals with long CAG repeat alleles at both *TCF4* and another locus, IRR pairs could sometimes originate from a locus other than *TCF4*. If such individuals were included in a GWAS of somatic expansion at *TCF4*, false positives would arise from variants tagging highly expanded alleles at other loci. To remove such effects while minimally impacting GWAS sample size, we applied a final, chromosome-specific sample exclusion filter: when testing variants on a given chromosome, we excluded individuals with anchored IRRs to loci on that chromosome (other than *TCF4*). We made one exception to this rule: we did not exclude individuals with anchored IRRs at *CA10*, as long *CA10* alleles are common but highly expanded *CA10* alleles are rare (Extended Data Fig. 1b).

4.3.3 GWAS meta-analysis of somatic repeat expansion in *TCF4*

We ran genome-wide association analysis independently in UKB (on our combined WGS+WES-derived *TCF4* somatic-expansion phenotype) and in AoU v7 (on our WGS-derived somatic-expansion phenotype) and then meta-analyzed the results.

In UKB, we adjusted for the following covariates: sex, age, age^2, 20 PCs, imputed allele length, age $\times$ imputed allele length, age^2 $\times$ imputed allele length, and coverage-adjusted *CA10* IRR count (i.e., $(\#_{\text{anchored IRRs at } CA10})/(\text{cov}_{\text{GC67}}/\overline{\text{cov}_{\text{GC67}}})$, which captured length variation in *CA10* alleles). We tested TOPMed-imputed variants [30] with MAF>0.1% using BOLT-LMM with the flag `--lmmForceNonInf` to require use of a non-infinitesimal linear mixed model [31,32].

In AoU, we adjusted for the following covariates: sex, age (at DNA acquisition; i.e., "biosample age"), age^2, 16 PCs, imputed allele length, age $\times$ imputed allele length, and age^2 $\times$ imputed allele length. We tested variants in the ACAF threshold srWGS joint callset (short-read WGS SNP and indel variants with population-specific allele frequency $>$1% or population-specific allele count $>$100 in any ancestry) using linear regression as implemented in BOLT-LMM.

We then meta-analyzed GWAS results from UKB and AoU using METAL [33]. To account for the additional power provided in the UKB GWAS by BOLT-LMM's non-infinitesimal mixed model, we computed standard errors of effect size estimates as $|\beta|/\sqrt{\chi^2_{\text{non-inf}}}$ (as β and standard

errors in the BOLT-LMM output are from the infinitesimal mixed model). To account for the different scale of the GWAS phenotypes (i.e., predicted age) in UKB versus AoU, we additionally scaled effect sizes and standard errors by the standard deviation of the GWAS phenotype within each cohort. We then performed meta-analysis, weighting according to these updated standard errors, and restricting to variants with minor allele frequency $>1\%$ in both cohorts.

We then extended our analysis to low-frequency coding variants. We examined the associations we had computed in UKB for all non-synonymous coding variants listed in the UKB WES "helper files" that had MAF between 0.1% and 1%. One variant associated with *TCF4* somatic expansion at a Bonferroni-significance level of p<0.05/23654: a missense variant in *MSH3* (chr5:80813660 T>G: *MSH3* Leu911Trp, $p = 1.5 \times 10^{-7}$). Finally, we extended the meta-analysis to low-frequency coding variants specifically at loci identified from the common-variant GWAS meta-analysis. Specifically, we identified 67 coding variants with a high or moderate impact VEP score [34] according to the AoU variant annotation table that were within 250kb of a lead variant (Table 1) and that had MAF between 0.1% and 2%. Four such variants associated with *TCF4* somatic expansion at a Bonferroni-significance level of p<0.05/67: two missense variants in *FAN1* that have previously been associated with Huntington's disease age-at-onset [35] (chr15:30910758 G>A: *FAN1* Arg507His, $p = 6.6 \times 10^{-11}$; and chr15:30905792 C>T: *FAN1* Arg377Trp, $p = 1.0 \times 10^{-5}$), a missense variant in *MSH3* (chr5:80813660 T>G: *MSH3* Leu911Trp, $p = 5.7 \times 10^{-6}$), and a missense variant in *OCM* that probably tags *PMS2*-related variation (chr7:5882598 G>T, $p = 1.7 \times 10^{-4}$).

5 Somatic repeat instability beyond CAG repeats

5.1 Extracting WGS reads derived from long STRs

To broaden the scope of our analyses to STRs beyond CAG repeats, we needed a way to ascertain WGS reads derived from long STR alleles that was efficient enough to run on hundreds of thousands of genomes. Unlike in the case of CAG repeats, we could not rely on such reads being mapped to a single locus (*TCF4*) or any small set of loci, so we instead implemented an algorithm to rapidly scan WGS alignments (cram files) and extract "highly repetitive" reads (as defined below) likely to be derived from long STR alleles.

The approach that we implemented was similar to ExpansionHunter Denovo (EHdn) [36] with a few optimizations to improve computational efficency. Like EHdn, we scanned all reads (regardless of mapping location) for evidence of periodicity: i.e., for a given period k, we counted the number of read positions i for which the bases at positions i and $i + k$ did not match. We considered a read to be "highly repetitive" if it had at most 10 mismatches for some $k \in \{2, 3, 4, 5, 6\}$. For each such read, we recorded the read and its alignment information (including where its mate

aligned). Limiting the motif length ($k \leq 6$) and capping the number of mismatches at 10 allowed us to implement an early-exit optimization: every 8 bases, we checked the numbers of accrued mismatches for each possible k, and if all of the mismatch counts exceeded 10, we skipped processing the remainder of the read. Additionally, we used the HTSlib API [37] to decode only the minimal set of cram fields necessary. These optimizations enabled us to run a streaming scan of a 30x WGS file (from a remote URL) in $\sim$8 minutes on the smallest UKB-RAP compute node (2 vCPUs), representing a $\sim$5x speedup over EHdn.

This tool typically extracted on the order of $\sim$10,000 reads per WGS sample that met the mismatch threshold ($\leq$10 mismatches between i and $i + k$) for one or more motif lengths k, excluding homopolymer repeats (as also done by EHdn). From this set of "highly repetitive" reads, we then performed further processing to identify IRRs (as described below; we used a slightly different definition of IRR compared to our CAG analyses, now that repeat motifs could have different lengths). We verified on a randomly selected set of 50 UKB WGS cram files that all IRRs reported by EHdn that satisfied our requirements for downstream analysis (see below) were also found by our algorithm.

5.2 Identifying IRRs anchored to their repeat loci of origin

To identify IRRs that could be anchored to known STR loci based on mate mapping locations, we performed follow-up analysis of the highly repetitive reads nominated by the above algorithm, applying the following steps:

1. **Definition of a set of known polymorphic STRs.** We primarily used the high-quality STR reference panel generated by Ziaei Jam et al. [7]. We downloaded both per-locus summary statistics (including coordinates and repeat unit sequence) and population-specific statistics (including allele frequencies and heterozygosity) from `https://github.com/gymrek-lab/EnsembleTR`. We then identified the subset of autosomal 2–6bp repeats (removing homopolymers listed with a longer motif, e.g., AA) that were polymorphic within the 1000 Genomes EUR population [8]. Finally, we augmented this set with 18 known disease-associated STRs found in the STRipy database [38] that were missing from this reference panel, resulting in a total of 356,131 autosomal repeats.
2. **Initial filtering of highly repetitive reads.** We dropped duplicate reads, reads for which the mate was unmapped or mapped to a 100kb bin containing no polymorphic STRs in the above set, and reads for which the mate was also highly repetitive (as we focused downstream analyses on anchored IRRs rather than IRR pairs).
3. **Identification of highly repetitive reads anchored to a known STR.** For each highly repetitive read that passed filtering and whose mate mapped $\geq$2bp away, we searched for known STRs within 5kb of the mate's mapping location (searching among the polymorphic STR

loci in the mate's 100kb bin). Among these potential STRs of origin, we identified those with a motif that was compatible with the highly repetitive read (using a simple first-pass filter of the motif appearing in the read greater than 70, 45, 32, 25, or 20 times for di-, tri-, tetra-, penta-, and hexa-nucleotide motifs, respectively). If more than one potential STR locus of origin passed this filter, we assigned the read to the STR locus closest to the mate.

4. **Identification of IRRs based on Hamming distance to pure repeats.** For each highly repetitive read that was anchored to a known STR locus, we computed its Hamming distance to repeat sequences comprised of the known STR motif (considering all possible cyclic rotations of the motif and its reverse complement), and we classified the read as an "IRR with Hamming distance $\leq X$" if it had Hamming distance at most 2, 3, or 4 to a 151bp pure repeat sequence. We also considered a stricter IRR definition that disallowed repeat interruptions (requiring 145bp of pure repeat sequence) but found the Hamming distance-based definitions to provide better statistical power.
5. **Restricting to well-anchored reads.** Among highly repetitive reads anchored to a known STR, we further required the mate had a mapping quality ≥ 30.
6. **Tabulation of observed reads per STR locus.** For each polymorphic STR with at least one well-anchored highly repetitive read, we computed the number of highly repetitive reads, number of strict IRRs ($\geq$145bp of pure repeat sequence), and number of IRRs with Hamming distance $\leq$ 2, 3, or 4 (restricting to well-anchored reads in all cases).

5.2.1 Assessing the effect of restricting to known polymorphic STR loci

Restricting analysis to IRRs that anchored to STR loci from a reference panel (primarily ref. [7]) drops some reads derived from repeats that are complex or difficult to map, so we assessed the effect of our choice to use an STR reference panel. First, for 100 randomly selected UKB WGS cram files, we examined all highly repetitive reads with Hamming distance $\leq$ 4 to a pure repeat sequence that were potentially mappable (based on the mate mapping $\geq$2bp away with mapping quality $\geq$ 30). Across these individuals, 15% of such reads were captured by our pipeline using the STR reference panel. Although this fraction seems low, closer inspection of the "missing" reads showed that they were unlikely to be amenable to short-read analysis. Many of these reads anchored to the same set of genomic regions that consistently contributed "missing" reads across samples. Among the 10 most missed repeat loci, 5 were located in difficult genomic regions (centromere, telomere, non-autosomal), and the other 5 were long in GRCh38 (>200bp for 4 repeats; 128bp for the remaining repeat), such that short-read data would be unlikely to permit robust analysis of these loci.

We also performed a reference-free pilot analysis searching for genomic loci at which counts of anchored highly repetitive reads associated with age, suggesting somatic repeat expansion. We identified 6,179 1kb bins for which at least 1% of the UKB cohort had a highly repetitive read

anchored to the given bin or one of its two flanking 1kb bins. Again, among these bins, only 1,104 ($\sim$18%) were represented in the STR reference panel, but the bins that were not represented tended to be in difficult genomic regions. All bins for which read count associated with age (at Bonferroni significance) were represented within the STR reference panel.

These two analyses suggest the reads that were lost by restricting to an STR reference panel predominantly originated from loci that were not amenable to short-read analysis, and that using a reference-based approach did not substantially hinder our ability to analyze somatic mutability.

5.3 Determining repeat loci with evidence of somatic mutability

Our IRR extraction pipeline identified 154 repeat loci for which long alleles were common: i.e., at least 2,500 UKB EUR individuals had at least one anchored IRR with Hamming distance $\leq$ 3 (Supplementary Data 1). We examined these 154 STRs for evidence of somatic instability by testing counts of anchored IRRs at these loci for association with age or with the genotypes of the variants in mismatch repair genes *MSH2* and *MSH3* that most strongly associated with somatic expansion in blood of *HTT* repeats [39] and *TCF4* repeats. We included the *MSH2* and *MSH3* genotypes in these tests based on pilot analyses (detailed in Section 5.3.1) that showed that among 10 STRs for which somatic-expansion phenotypes associated with age, for some STRs, the *MSH2* or *MSH3* genotype associated more strongly with IRR count than did age (providing increased power to identify evidence of somatic instability), presumably because for these STRs, the cumulative effects of *MSH2* or *MSH3* variants on somatic instability over an individual's years of life (mean $\sim$57 years in UKB) were easier to discern than the effect of individuals having experienced somatic mutation for differing numbers of years of life (s.d. $\sim$8 years).

In more detail, to maximize power to detect associations with counts of anchored IRRs, we first adjusted these measurements for sequencing coverage and inherited allele length. For each of the 154 STRs, for each of two IRR definitions (Hamming distance $\leq$ 3 and $\leq$ 4), we applied the following procedure:

1. Divide each individual's IRR count by the individual's WGS coverage.
2. Estimate each individual's inherited allele lengths (up to a scale factor). We did so by imputing coverage-adjusted IRR counts within the UKB cohort. Specifically, for each of an individual's two haplotypes at the STR locus, we identified the 10 longest haplotype matches in the cohort ("haplotype neighbors"). We then ran 20 phasing iterations in which we cycled through individuals, phasing each individual's coverage-adjusted IRR count by partitioning it according to the ratio of the mean of the phased coverage-adjusted IRR counts of haplotype 1's neighbors to the mean of haplotype 2's neighbors. In the final phasing iteration, we reported these two means as the individual's imputed allele lengths (up to a scale factor).

3. Adjust coverage-adjusted IRR counts for imputed values (summed across each individual's two haplotypes): i.e., run linear regression and compute the residual.

4. Crop the residuals to limit the fraction of variance contributed by outliers. Specifically, we cropped at ±s.d.(resid) × $\sqrt{n/100}$.

We ran the above pipeline on n=421,364 unrelated UKB participants of EUR genetic ancestry (dropping one individual per second-degree or closer relationship pair, i.e., kinship >0.0884 as computed by UKB [1]). For each of the 154 STRs, for each of the two IRR definitions (Hamming distance ≤ 3 and ≤ 4), we then tested the adjusted IRR counts for association with *MSH2* (chr2:47416318 G>A), *MSH3* (chr5:80638411 G>T), and age in a joint model also including 10 genetic PCs and sex. A repeat locus was considered to have evidence of somatic instability if one or both of its adjusted IRR count measurements had a significant ($p < \frac{0.05}{154 \cdot 3 \cdot 2} \approx 5.4 \times 10^{-5}$) association with *MSH2*, *MSH3* or age. For each of the 17 loci that were found to be somatically unstable, we recorded the Hamming distance threshold (3 or 4) that resulted in the most significant association and used this threshold in downstream analyses (Supplementary Data 2).

Associations of IRR counts at an STR (proxying for repeat length) with *MSH2* or *MSH3* genotype could in theory capture effects of these variants on germline mutation rather than somatic mutation. However, we concluded that this was unlikely based on multiple lines of evidence. First, the 17 STR loci at which we detected associations with either *MSH2* or *MSH3* genotype were located in *trans* to these mismatch repair genes, usually on different chromosomes, such that an effect of one of these modifier variants on germline mutation of an STR would only have acted on an observed allele's length for an average of one generation. As such, a germline explanation of these associations would require an extremely large, directional effect of *MSH2* or *MSH3* variation on germline mutation of an STR, which has not previously been observed [11]. Second, the 17 STR loci generally showed evidence of repeat expansion with age: for 10 of the 17 STRs, we observed a significant association of adjusted IRR count with increased age, and for 15 of the 17, we observed a positive trend with age; the negative trends for the remaining two STRs were not significant ($p > 0.05$) (Supplementary Data 2). Third, our genome-wide association analyses of IRR count-based measurements produced results broadly concordant with GWAS of direct observations of somatic expansion of AAAG repeats (described below; Fig. 3a).

Finally, to rule out the possibility that our observed evidence of somatic mutations was confounded by clonal expansions in blood, we tested whether adjusted IRR counts at any of the 17 loci, or somatic expansion of short CAG repeat alleles in *GLS*, *ATN1*, *TCF4*, and *DMPK*, were associated with mosaic chromosomal alterations (mCAs), mosaic X or Y chromosome loss [25], or telomere length. No associations were Bonferroni-significant (minimum p-value 0.002 for $21 \times 4 = 84$ tests; Bonferroni-adjusted p-value of 0.17).

5.3.1 Pilot analyses motivating using association with *MSH2* and *MSH3* haplotypes to find unstable STRs

The approach described above, in which we searched for evidence of somatic instability of STRs by testing somatic-expansion phenotypes for association with age or with *MSH2* or *MSH3* haplotypes, was motivated by a pilot analysis in which we initially identified somatically unstable STR loci as those for which repeat length (measured by IRR count) associated significantly with age. This analysis (which looked only at age, not at association with *MSH2* or *MSH3* variants) identified 10 somatically unstable STRs (p-value for age < 0.00016; Fig. 3a). When we ran GWAS on somatic expansion of these 10 STR loci, we observed that eight of the 10 STRs had at least one GWAS hit, and for all eight STRs, the lead variant in the GWAS was either at *MSH2* or *MSH3* (Extended Data Fig. 8 and Supplementary Data 3). Moreover, for half of the STRs, the somatic expansion phenotype associated more strongly with the lead GWAS variant than it did with age. This suggested the possibility of identifying a few additional somatically unstable STRs by also ascertaining unstable repeats based on their association with genetic variants at mismatch repair genes.

To minimize hypothesis testing burden and computational cost, we focused this additional identification strategy on *MSH2* and *MSH3* haplotypes (based on the above observation that for all of the somatically unstable STRs identified from the age association analysis, the lead GWAS locus was either *MSH2* or *MSH3*). We further observed that at *MSH2*, the G322D missense variant chr2:47416318:G>A always had the strongest or near-strongest signal (>90% the strength of the lead variant), and at *MSH3*, the common haplotype chr5:80638411:G>T was always a good representative of the locus (lead variant for three STRs; always ≥50% the strength of the lead variant). We therefore tested all of the 154 STRs for association with these two variants, identifying an additional seven STRs with evidence of somatic instability (for a total of 17 somatically unstable STRs, including the 10 initially identified based on association with age).

While this approach could in theory bias our ascertainment of unstable STRs to those affected by *MSH2* and *MSH3* variants, this bias is at most modest (given that 10 of the 17 unstable STRs were identified based on association with age alone) and probably minimal (given that *MSH2* or *MSH3* was the lead GWAS locus for all of the age-identified unstable STRs).

5.4 GWAS on somatic expansion of long repeat alleles

For the 17 repeat loci for which adjusted IRR counts showed evidence of somatic repeat instability, we performed GWAS on unrelated UKB participants of European genetic ancestry (n=419,013). In more detail, for each STR, for the Hamming distance threshold selected in the previous analysis, we adjusted IRR counts for sequencing coverage and inherited allele length as described above, and in the final step, we cropped the residualized coverage-adjusted IRR measurements more strin-

gently (at $\pm$s.d.(resid) $\times \sqrt{n/200}$) to improve robustness of association test statistic calibration for low-frequency variants. BOLT-LMM was then used to computed linear regression association statistics for all TOPMed-imputed variants with MAF $> 1\%$ and INFO score > 0.8, adjusting for sex, age, age^2, imputed allele length sum, interactions between imputed allele length sum with both age and age^2, and 20 genetic PCs. Manhattan plots are shown in Extended Data Fig. 8; lead variants that are Bonferroni-significant ($p < 5 \times 10^{-8}/17$), excluding variants <5Mb from the repeat locus being tested, are provided in Supplementary Data 3.

5.5 Local heritability at DNA repair genes

For the same 17 STRs, we estimated the contribution of genetic variation to their adjusted IRR count phenotypes (i.e., local heritability of these phenotypes) from five loci containing DNA repair genes implicated in repeat expansion (*MSH2*, *MSH3*, *PMS2*, *FAN1*, and *MLH3*). We ran BOLT-REML [40] on all SNP-array variants within 1Mb (500kb on either side) of chr2:47416318 for *MSH2*, chr5:80638411 for *MSH3*, chr7:6003481 for *PMS2*, chr14:74986336 for *MLH3* and chr15:30902611 for *FAN1*. We ran these analyses on unrelated UKB participants of European genetic ancestry. For *TCF4*, we substituted our better-powered somatic expansion phenotype from our *TCF4* expansion GWAS. We controlled for sex, age, age^2, and 20 genetic PCs. We ran BOLT-REML with the flag `--remlNoRefine`. No local heritability estimates were available for the chr2:62.7 AG repeat, as the heritability estimate was too close to 0. We computed the relative contribution of a given genetic modifier as the proportion of heritability attributed to the modifier locus (relative to the total heritability attributed to the five considered modifiers). We restricted this analysis to loci for which at least one of the five modifiers had a local heritability estimate with a z-score of at least 2.5 (i.e., $h_g^2/\text{s.e.}(h_g^2) > 2.5$). Estimates and standard errors are available in Supplementary Data 4.

5.6 Quantifying somatic expansion of mid-length AAAG repeat alleles

5.6.1 Identifying reads spanning AAAG repeats

Among the 17 STRs for which adjusted IRR counts exhibited evidence of somatic repeat instability, we identified two AAAG repeats (at chr19:14.8Mb and chr2:232.4Mb) for which mid-length alleles (containing 19–26 AAAG repeat units) were sufficiently common and sufficiently unstable to directly observe and analyze somatic repeat expansions using spanning reads. To do so, for each repeat locus, we extracted reads aligned to the locus, mates that aligned elsewhere in the genome, and mates of those mates (to recover read sequences of supplementary alignments; see Section 5.7.1) and among all of these reads, we identified spanning reads using the following approach (summarized below and detailed in the provided code):

1. Search for `AAAGAAAG` in the read sequence; if found, reverse-complement the read sequence (since both of the repeats that we studied are on the minus strand in GRCh38).

2. Find the longest substring consisting mostly of T or C bases with 4bp periodicity. To allow mismatches, we gave base i a score of 1 if it was a T or C and matched base $i + 4$; otherwise we gave base i a score of -2. We then identified the highest-scoring substring of the read sequence as the putative repeat sequence.

3. Require that the putative repeat sequence be $>$10bp long with $\geq$90% TC content and with C's comprising 1/3 to 1/5 of its bases.

4. Check flanking base pairs on the left and right of the repeat for consistency with the reference sequence, and adjust the span of the repeat sequence if necessary (as sequencing errors could cause the highest-scoring substring to have endpoints slightly offset from the repeat sequence). The general idea was to look for an exact 3bp match or a near-exact 5bp match (allowing one error) to flanking sequence, with additional logic to handle error modes.

5.6.2 Estimating mean somatic expansion of an individual's mid-length allele

Using spanning reads identified by the above approach, we identified individuals with evidence of a mid-length allele and computed a "mean somatic expansion" phenotype estimating the extent of somatic expansion of this allele (i.e., the mean across cells of the number of repeat units somatically gained) based on the length distribution of spanning WGS reads. Our overall approach to do so was to (1) call the inherited allele, (2) identify the set of spanning reads likely to be derived from this allele (possibly after somatic mutation), (3) compute the mean length of the alleles spanned by these reads, and (4) subtract the estimated length of the inherited allele. We implemented this approach differently for the two AAAG repeat loci (to optimize for their differing allele frequency distributions) as outlined below.

For the chr19:14.8Mb AAAG repeat (in *ADGRE2*), we defined allele length as the number of full repeat units, such that the GRCh38 reference allele `T(TTTC)`$_3$`TTTTT` has repeat length 3. The 3-repeat allele of this STR is common, as are long alleles that produce IRRs; mid-length alleles are less common. We therefore focused on identifying and phenotyping individuals heterozygous for one mid-length allele and another allele that was clearly longer or shorter. To do so, for each WGS sample, we looked for the shortest allele length L in the range $L \in \{17, 18, \ldots, 29\}$ satisfying either (i) L was supported by $\geq$2 spanning reads; or (ii) $L \geq 21$ and L was supported by one spanning read and $L + 1$ was supported by 1–2 spanning reads. (The second criterion helped handle cases in which alleles were so unstable that most cells contained a somatic expansion.) If such an allele L was found, we then looked for evidence of a clearly-distinguishable second allele (based on $\geq$3 supporting reads): a shorter allele, an allele interrupted by an indel, or a much

longer allele ($\geq$7 repeats longer; we included flanking reads and IRRs as supporting reads). If we observed evidence of a second allele, we considered the individual to have inherited one copy of the allele L. We then tabulated spanning reads of other lengths putatively derived from this allele: $L + 1$, $L + 2$, ..., $L + 7$ (stopping upon encountering three consecutive allele lengths with no spanning read support), and $L - 1$ (which rarely had a spanning read, such that we treated such reads as observations of the allele L under the assumption that a PCR stutter error was the most likely explanation). Finally, we computed the individual's "mean somatic expansion" as the mean allele length represented in this distribution, minus L.

For the chr2:232.4Mb AAAG repeat, we defined allele length as the number of full repeat units plus 1, such that the GRCh38 reference allele $\texttt{(TTTC)}_{16}\texttt{TTTTTTTT}$ has repeat length 17. Short and mid-length alleles of this STR are common, whereas long alleles are much less common. We therefore focused on identifying and phenotyping individuals heterozygous for two distinct short or mid-length alleles supported by spanning reads. For each WGS sample, we first looked for the shortest allele length $L_1 \leq 29$ satisfying either (i) L_1 was supported by $\geq$3 spanning reads; or (ii) $L_1 \geq 21$ and L_1 was supported by 2 spanning reads. We allowed L_1 to have a non-integer length (corresponding to an allele with an indel interruption). If a value of L_1 satisfying (i) or (ii) was found, we then looked for a longer allele length $L_2 > L_1$, supported by $\geq$2 spanning reads, for which L_2 had more supporting reads than $L_2 - 1$ and $L_2 - 2$ combined. We relaxed the latter requirement in scenarios in which L_1 and L_2 were more easily distinguished, e.g., if one of L_1 or L_2 was interrupted, or if $L_1 \leq 21$ such that it was expected to exhibit a low amount of somatic expansion. If L_1 and L_2 were successfully identified, we then measured the "mean somatic expansion" of L_2 in the same way as for the chr19:14.8Mb repeat.

5.6.3 Differences in approach compared to spanning-read analyses of CAG repeats

The above approach differs from the approach that we developed to estimate somatic instability of CAG loci by analyzing spanning reads (Fig. 1d and Extended Data Figs. 3 and 4) because the CAG and AAAG repeats turned out to have very different relative signal-to-noise ratios of somatic expansion versus PCR stutter:

- For CAG repeats, we had observed that PCR stutter errors comprised a considerable fraction of apparent repeat expansions, which were infrequently observed (Supplementary Fig. 3b). As such, to accurately quantify instability of CAG repeats, we needed to stringently filter for PCR stutter error (by analyzing base qualities within reads), considerably reducing the pool of reads available for analysis in order to minimize bias in our estimates of instability.

- In contrast, the mid-length alleles of the chr19:14.8Mb and chr2:232.4Mb AAAG repeats are highly mutable, such that reads containing somatic expansions were usually observable in carriers of alleles with 22+ repeats, and few of these reads could have originated

from PCR stutter based on the observed relationship with age (Fig. 4d and Extended Data Fig. 10a,b). Additionally, a main focus of our analyses of mid-length AAAG repeat instability was to identify germline drivers (by generating a powerful GWAS phenotype) rather than to compute an unbiased estimate of mutation rate. This motivated retaining as many reads as possible for analysis rather than attempting to filter for PCR stutter.

5.6.4 Complexities of assessing instability of mid-length alleles of other STRs

We considered attempting similar analyses of mid-length alleles for other STRs among the 17 repeat loci with IRR-based evidence of somatic instability, but we found that most of the other STRs did not have common uninterrupted mid-length alleles based on HGSVC3 assemblies [41]. Additionally, some had common polymorphisms in the base pairs flanking the repeat that complicated genotyping. We attempted a pilot analysis of mid-length alleles of the highly age-associated chr15:74.8Mb AC repeat but observed that unlike the two AAAG repeats, this repeat appeared to be strongly affected by PCR stutter artifacts, precluding confident ascertainment of somatic expansions. Further method development will be needed to enable broader analyses of somatic mutation of mid-length STR alleles and address the following challenges:

1. The mid-length alleles of an STR that provide the most information about somatic instability are typically the longest, most mutable alleles, but these alleles also have the fewest spanning reads (due to their length). As such, the precise criteria used to identify spanning reads (e.g., thresholds on numbers of flanking bases and mismatches allowed) can substantially impact performance, and the parameter values that maximize detection sensitivity while controlling false positives are locus-specific.

2. The germline allele from which a putatively-expanded allele originates can be difficult to determine. In the simplest scenario, an individual is heterozygous for two distinct germline alleles, each supported by several spanning reads. However, in instances in which only one germline allele has support from multiple spanning reads, the individual is likely to either be homozygous for a single germline allele, heterozygous for a mid-length allele that has expanded to many different lengths, or heterozygous for a long allele. These possibilities can usually be distinguished based on counts of spanning reads, flanking reads, and IRRs, but the optimal way to utilize this information depends on the allele frequency distribution, level of instability, and error profile of a specific locus.

3. The sequence immediately flanking a repeat can harbor common variants that need to be taken into account when identifying spanning reads.

4. The extent to which PCR stutter error contributes to observed spanning reads supporting putative expansions is locus-specific, such that filtering may or may not be required.

5.7 Refined GWAS on somatic expansion of AAAG repeats

5.7.1 Optimizing somatic-expansion phenotypes of AAAG repeats for GWAS

Given the strong associations of age and *MSH3* genotypes with somatic expansion of long AAAG repeats at chr19:14.8Mb (using the adjusted IRR count phenotype) and with somatic expansion of mid-length AAAG repeats at both chr19:14.8Mb and chr2:232.4Mb (using the mean somatic expansion phenotype), we sought to optimize these phenotypes for further genome-wide association analyses. To do so, we determined transformations of these phenotypes that increased statistical power to detect associations and also increased robustness of rare variant association test statistics (by pulling in outlier values in the phenotype distribution).

For somatic expansion of long chr19:14.8Mb AAAG alleles, we generated a predicted age phenotype (similar to the approach we used for optimizing the *TCF4* somatic-expansion phenotype). In more detail, we first performed a more comprehensive search for IRRs derived from this commonly-expanded repeat: because the GRCh38 reference only contains a 3-repeat allele, IRRs with mates containing several repeat units sometimes anchored to the locus with low mapping quality or only had a supplementary alignment to the locus. We therefore performed a targeted search for reads originating from this locus by (1) extracting reads aligned to the locus (with any mapping quality, and including supplementary alignments); (2) extracting mates that mapped elsewhere in the genome; and (3) extracting mates of such mates (to recover the read sequences of reads that had been hard-clipped in supplementary alignment records). From this set of reads, we identified IRRs with Hamming distance ≤ 4 to a pure AAAG repeat sequence. We then adjusted these IRR counts for sequencing coverage and inherited allele length (based on imputation) as before, cropping the residuals at $\pm$s.d.(resid) $\times \sqrt{n/200}$. Finally, we evaluated how age varied (on average across samples) as a function of these optimized adjusted IRR counts, observing a sigmoid relationship. As such, we fit the model

$$\text{age} \sim a + \frac{k - a}{1 + q \times \exp(-b \times \text{adjusted IRR count})} \tag{4}$$

in `R` using `nls()`. We then transformed the adjusted IRR count of each individual to the "predicted age" scale according to this model fit, obtaining our optimized chr19:14.8Mb (*ADGRE2*) long-allele somatic-expansion phenotype.

To generate high-powered GWAS phenotypes from our "mean somatic expansion" measurements of chr19:14.8Mb and chr2:232.4Mb AAAG mid-length alleles, we used a different approach to adjust for the strong effects of age and inherited allele length on these measurements. The intuition behind our approach was to convert an individual's observed age, inherited allele length, and mean somatic expansion into an estimate of that individual's genetic liability for somatic expansion. To accomplish this, we took the following approach to generate optimized mid-

length somatic-expansion phenotypes for each of the two AAAG repeat loci, independently in UKB and AoU (v8). We restricted analysis to allele lengths between 19 and 26 (which were long enough to be unstable but short enough to allow confident genotyping of somatic expansions), and we generated optimized somatic-expansion phenotypes separately for the allele lengths $19, 20, 21, 22, 23, \{24, 25, 26\}$ (i.e., we grouped together the three lower-frequency alleles 24–26 for analysis). For each allele length category, we fit a model that tried to predict an individual's genotype g for the lead *MSH3* variant (chr5:80638411:G:T)—which served as a proxy for the individual's genetic liability—from the individual's observed mean somatic expansion rate per year of life:

$$\text{per-year somatic expansion rate } r = \frac{\text{mean somatic expansion}}{\text{age}}. \tag{5}$$

Empirical examination of the relationship between g (averaged across many individuals) and r suggested fitting the following model for the expected value of g given r:

$$\overline{g}(r) = \begin{cases} g_0 & \text{if } r = 0 \\ a \cdot \log r + c & \text{if } r > 0. \end{cases} \tag{6}$$

We fit this model by computing g_0 as the mean value of g among individuals in the allele length category with $r = 0$ (i.e., no observed somatic expansion) and computing best-fit values of a and c among individuals with $r > 0$. Finally, after fitting this model separately for each allele length category, we applied the fitted models to convert each individual's observed age, inherited allele length, and mean somatic expansion into an estimated genetic liability for somatic expansion, which we used as our GWAS phenotype. Because this set of models contained a small number of parameters to be optimized (3 parameters each for 6 allele length categories), overfitting was not a concern. To confirm this, we verified that substituting a different variant to use as a proxy for genetic liability had a negligible impact on the transformation.

5.7.2 GWAS of AAAG somatic-expansion phenotypes in UKB and AoU (v8)

In UKB, we ran three genome-wide association analyses on our optimized somatic-expansion phenotypes for chr19:14.8Mb AAAG long alleles, chr19:14.8Mb AAAG mid-length alleles, and chr2:232.4Mb AAAG mid-length alleles. In all of these GWAS we restricted to individuals with EUR genetic ancestry, and in the analyses of mid-length alleles we restricted to individuals heterozygous for a mid-length allele of length 19–26. GWAS sample sizes are provided in Supplementary Data 5. For chr19:14.8Mb AAAG long alleles we adjusted for the following covariates: sex, age, age^2, 20 PCs, imputed allele length sum, and interactions between imputed allele length sum with both age and age^2. For mid-length alleles we adjusted for the following covariates: sex, age, age^2, 20 PCs, inherited allele, inherited allele^2, age $\times$ inherited allele, age^2 $\times$ inherited allele,

age $\times$ inherited allele^2, and age^2 $\times$ inherited allele^2. We tested TOPMed-imputed variants [30] with MAF>0.1% using BOLT-LMM with the flag `--lmmForceNonInf` to require use of a non-infinitesimal linear mixed model [31, 32]. We additionally tested gene burden masks that collapsed together variants predicted to cause loss of function (pLoF) and optionally included missense variants satisfying PrimateAI-3D score thresholds [42] (>0.6, 0.7, 0.8) as recently described [43]. For downstream analyses of effect sizes of pLoF variants, we used the burden masks composed of pLoF SNPs and indels (in MANE select transcripts [44]) in the UKB WGS DRAGEN variant call set and CNVs that we previously called from UKB WES data [29], applying an allele frequency threshold of < 0.0001 to all variants.

In AoU (v8), we ran four genome-wide association analyses on our optimized somatic-expansion phenotypes for chr19:14.8Mb AAAG mid-length alleles, restricting to individuals with EUR genetic ancestry, and chr2:232.4Mb AAAG mid-length alleles, restricting to individuals with EUR genetic ancestry, with AFR genetic ancestry, or with AMR genetic ancestry. We restricted to individuals heterozygous for a mid-length allele of length 19–26; GWAS sample sizes are provided in Supplementary Data 5. We adjusted for the same covariates as in our GWAS of mid-length-allele somatic expansions in UKB, with the exception of using 16 PCs (in the AoU v8 data release) rather than 20 PCs. We tested variants in the ACAF threshold srWGS joint callset (short-read WGS SNP and indel variants with population-specific allele frequency $>1\%$ or population-specific allele count >100 in any ancestry) using BOLT-LMM with the flag `--lmmForceNonInf` to require use of a non-infinitesimal linear mixed model [31, 32]. We then restricted to variants with MAF $< 0.1\%$ in the association tests.

The AoU v8 data release (n=414K WGS) included a small fraction of samples that were derived from saliva (12%) rather than blood. We decided to include these samples in GWAS given that human DNA derived from saliva has been estimated to be ~80–90% leukocyte-derived [45], to the extent that clonal hematopoiesis mutations are observed in saliva at nearly the same variant allele frequencies (VAFs) as in blood from the same individuals (making saliva and blood essentially interchangeable for analysis of low-VAF somatic mutations in blood) [46]. Consistent with these observations, we observed broadly concordant effect sizes of GWAS hits in blood and saliva, validating the approach of including all samples in the GWAS to maximize power.

5.8 Fine-mapping GWAS signals and computing polygenic scores

For the well-powered GWAS of somatic expansion of chr19:14.8Mb AAAG long alleles and chr2:232.4Mb AAAG mid-length alleles, we fine-mapped GWAS loci using susieR [47] in UK Biobank. In more detail, we first extracted lead variants from these analyses, obtaining a set of 10 variants associated with somatic expansion of chr19:14.8Mb AAAG long alleles and 13 variants for chr2:232.4Mb AAAG mid-length alleles. For each lead variant, we extracted TOPMed-imputed SNP and indel variants within 2Mb (1Mb on each side) of the lead variant with an association

significance of $p < 0.01$. If more than 3,000 such variants existed, we restricted to the top 3,000 most significant variants. We restricted to individuals of European genetic ancestry and regressed out the relevant covariates (i.e., all covariates included in our refined GWAS described above). We then ran SuSiE allowing up to 10 nonzero effects in the regression model. For each locus, for each identified causal set, we report the lead variant within the set and the number of variants within the set in Supplementary Data 6. For four loci near the genome-wide significance threshold ($p = 6.5 \times 10^{-9}$ to 4.0×10^{-8}; two each for the two GWAS), SuSiE did not identify any causal sets.

We used these fine-mapped variants to train a polygenic score for somatic expansion of mid-length alleles of the chr2:232.4Mb AAAG repeat in UKB. We estimated polygenic score coefficients by fitting a joint model predicting our optimized somatic-expansion phenotype using all lead variants from all identified causal sets (Supplementary Data 6). We then used these coefficients to compute polygenic scores in AoU (v8) and explore variability in somatic expansion rates across individuals in different polygenic score percentiles (Fig. 4d).

5.9 Estimating relative expansion rates of groups of individuals

To compute the relative expansion rates of chr2:232.4Mb AAAG mid-length alleles in individuals with different pairwise combinations of genotypes at top modifier loci (*MSH3*, *MSH2*, and *MLH3*; Fig. 4e) relative to the rate in the overall population, we applied the following approach to AoU (v8) participants with European genetic ancestry:

- For each mid-length repeat allele length $L \in \{19, 20, 21, 22, 23\}$:
  - Estimate the population-wide expansion rate $\hat{\beta}_{\text{pop}}$ (and its standard error) from a simple linear regression of the "mean somatic expansion" phenotype on age, including all European-ancestry individuals with a somatic expansion measurement of the allele L.
  - Estimate the within-group expansion rate $\hat{\beta}_{\text{group}}$ (and its standard error) in an analogous manner, restricting to individuals in the group of interest (e.g., individuals with one copy of the common *MSH3* eQTL and no copies of the low-frequency *MSH2* missense variant).
  - Compute the relative expansion rate (within the group vs. population-wide) as the ratio of $\hat{\beta}_{\text{pop}}/\hat{\beta}_{\text{group}}$ and estimate the confidence interval of this ratio using Fieller's theorem (`twopartm::FiellerRatio`). We approximated the variance of the ratio (assuming minimal covariance between β_{pop} and β_{group}) using the delta method:

$$\frac{1}{\hat{\beta}^2_{\text{pop}}} \times \left(\hat{\sigma}^2_{\text{group}} + \left(\frac{\hat{\beta}_{\text{group}}}{\hat{\beta}_{\text{pop}}} \right)^2 \hat{\sigma}^2_{\text{pop}} \right),$$

where each $\hat{\sigma}^2$ is the estimated variance of the corresponding $\hat{\beta}$.

- The above procedure produced separate estimates of relative expansion rates and their standard errors for each of the mid-length allele lengths $\{19, 20, 21, 22, 23\}$ (selected for being the most informative). To obtain a single combined estimate and 95% confidence interval, we performed random-effect meta-analysis (`meta::metagen` package; effect estimate is `TE.random`; confidence interval is `lower.random` and `upper.random`).

We applied an analogous approach to estimate the relative somatic expansion rates of mid-length chr2:232.4Mb AAAG repeat alleles in current smokers versus never-smokers in UKB, restricting these analyses to unrelated European-ancestry participants.

5.10 GWAS meta-analysis for mid-length AAAG repeat expansion

To maximize statistical power to detect inherited variants associated with somatic repeat expansion of chr19:14.8Mb and chr2:232.4Mb AAAG mid-length alleles, we meta-analyzed the European-ancestry GWAS results (independently for the two AAAG repeat loci) we had computed in UKB and AoU (v8) using METAL [33]. To account for the additional power provided by BOLT-LMM's non-infinitesimal mixed model, we computed standard errors of effect size estimates as $|\beta|/\sqrt{\chi^2_{\text{non-inf}}}$ (as β and standard errors in the BOLT-LMM output are from the infinitesimal mixed model). We then performed meta-analysis, weighting according to standard errors, and restricting to variants tested in both cohorts, with a MAF>0.1% in the association tests within both cohorts, and with a similar MAF between the cohorts (within a factor of 2).

To report effect sizes on the standard deviation scale, we divided effect sizes from the meta-analysis by the standard deviation of each optimized somatic-expansion phenotype in UKB (0.0073 for chr19:14.8Mb mid-length alleles, 0.0034 for chr2:232.4Mb mid-length alleles). We verified that the standard deviations of the corresponding phenotypes in AoU (v8) were similar (0.0071 and 0.0027, respectively).

5.11 Comparison of genetic modifier effects across repeat loci and HD phenotypes

To explore the extent to which inherited genetic modifiers of somatic repeat expansion acted similarly or differently on expansion of different STR loci, we created a shortlist of such modifier haplotypes and examined their associations with a shortlist of repeat expansion phenotypes (Fig. 3c).

To create the shortlist of inherited genetic modifier haplotypes, we drew from our well-powered GWAS of AAAG repeat expansions and the *TCF4* CAG repeat expansion as well as a recent well-powered GWAS of *HTT* CAG repeat expansion in blood [39]. Among the GWAS loci found by

these analyses, we identified 13 loci containing genes involved in DNA repair and DNA damage response, and we selected 1–3 independently associated variants per locus to include in our shortlist. For 10 of these loci (*PARP1*, *MSH2*, *MSH6*, *PMS1*, *XPC*, *SMARCAD1*, *MSH3*, *PMS2*, *NEIL2*, and *MLH3*), we selected variants representing fine-mapped credible sets from our GWAS of AAAG somatic repeat expansion (Supplementary Data 6), including both a common variant and a low-frequency missense variant when available. At *MSH3*, we also included a rare haplotype previously implicated in *HTT* repeat expansion in blood and HD age-at-onset (5ABEM2 / 5AM2 [39]), and we selected the lead *MSH3* eQTL haplotype (5ABEM1 / 5AM1) as the common *MSH3* variant in our shortlist. At *FAN1*, we selected one common and one missense variant (15ABEM1 and 15ABEM2) from the GeM-HD analysis [39]. Finally, at *GADD45A* and *ATAD5*, we selected two variants from our meta-analysis of somatic expansion of *TCF4* repeats in blood (Supplementary Table 4).

To create the shortlist of repeat expansion phenotypes, we identified 8 STRs (from among the 17 repeat loci with IRR-based evidence of somatic instability) for which at least 2 of the 20 selected variants had $p < 5 \times 10^{-8}$ in our GWAS of adjusted IRR counts in UKB (Section 5.4). We additionally included our optimized *TCF4* somatic-expansion phenotype, which satisified this criterion; we extracted association statistics from our meta-analysis in UKB+AoU (Section 4.3.3). Finally, we included the *HTT* blood-expansion phenotype (somatic expansion ratio; SER) and four Huntington's disease clinical onset phenotypes: hastening of age-at-onset, SDMT, TFC6, and TMS [39]. The summary association statistics for these phenotypes [39] did not include the low-frequency *PMS1* missense variant chr2:189795860, so we instead extracted its association statistics from an earlier analysis of similar clinical phenotypes: HD onset, SMDT30, TFC6, and TMS30 [48].

6 Phenotypic associations of long repeats

To search for potential effects of repeat expansions (inherited and/or somatic) on health-related phenotypes, we first generated repeat-expansion genotypes encoding the long-allele-carrier status of each UKB participant at 67,405 STR loci. We defined long-allele-carrier status as a binary genotype indicating the observation of at least one IRR with Hamming distance ≤ 3 to a pure repeat sequence (Section 5.2). We then identified 67,778 STRs for which >5 UKB EUR-ancestry participants carried a long allele; we further restricted the list to 67,405 STRs with a minor allele count $\geq$5 among unrelated EUR-ancestry individuals (n=421,364, which we used for association testing).

6.1 Associations with quantitative traits

We first tested long allele carrier status for association with 57 heritable quantitative traits that we previously curated [49] (including anthropometric traits, blood pressure, measures of lung function, bone mineral density, blood cell indices, and serum biomarkers). To reduce multiple hypothesis testing burden, we focused these analyses to $\sim$4,200 STRs for which long alleles were carried by at least 0.02% of individuals (MAF $> 10^{-4}$) in the association test (4,108–4,225 STRs depending on the phenotype); we determined that we were underpowered to detect quantitative trait associations with ultra-rare STR expansions. For each STR, we tested long-allele-carrier status for association with each of the 57 heritable quantitative traits using linear regression, adjusting for sex, age, age^2, and 20 genetic PCs as covariates. We identified 36 associations that reached a Bonferroni significance threshold of $p < 2.1 \times 10^{-7}$. We then filtered associations that could possibly be explained by linkage disquilibrium with SNP and indel variants by requiring each association to remain significant after conditioning on any other more-strongly associated SNP or indel within 2Mb, as described previously [9,49,50]. This filtering resulted in 23 remaining associations reported in Supplementary Data 7.

The 23 associations, which involved seven distinct STR loci, replicated several associations recently observed [51, 52] in analyses of an earlier UKB 200K WGS release. Quantitative trait associations at two STR loci had not previously been reported: *GLS*, which we followed up in detail (see below), and *RABGAP1*, at which long-allele status for an intronic repeat associated with reduced height (–0.08 (s.e. 0.01) standard deviations).

6.2 Associations with binary disease outcomes

We additionally tested long-allele-carrier status of all 67,405 STRs for association with 6,483 binary phenotypes that we defined as follows. We first extracted binary disease outcome data (as of 2025-04-01) that UK Biobank had curated from several sources (self-report, electronic health records, and cancer and death registries) and grouped according to ICD-10 categories (at 3-character or 4-character resolution):

- "First occurrence" data fields that UKB had generated (at the 3-character level) by merging cases across data sources
- Hospital episode statistics (field 41270)
- Cancer registry data (field 40006)
- Death registry data (fields 40001 and 40002)

For each ICD-10 category (at 3-character or 4-character resolution), we generated an "all source" phenotype (the union across the above) and an "HES/cancer/death" phenotype (the union across fields excluding the first occurrence field, to exclude self-reported cases). Among the all-source,

HES/cancer/death, HES-only, cancer-only, and death-only phenotypes (comprising up to five versions of each ICD-10 category), we identified phenotypes with at least 50 cases in the full UKB cohort. Finally, for each ICD-10 category, we de-duped these phenotypes using the following procedure:

- Always retain the phenotype constructed via the union of all sources (if it meets the minimum case count)
- Source-specific categories that meet the minimum case count are kept if they have $<$90% as many cases as the all-source category (such that they are not redundant with it) and meet one of the following criteria:
  - The category is the HES/cancer/death category
  - The HES/cancer/death category was deemed redundant ($\geq$90% of the all-source category) and was excluded
  - The category has $<$90% as many cases as the HES/cancer/death category

The above procedure generated a set of 6,483 de-duped binary disease phenotypes with a minimum case case count of 50 in UKB.

We tested long-allele-carrier status of each of the 67,405 STRs for association with each of the 6,483 binary phenotypes using the BinomiRare test [53] to obtain p-values robust to case-control imbalance while adjusting for age and sex. As previously described [9, 49, 50], for computational efficiency, we reimplemented the BinomiRare test and applied a binomial approximation when the number of observed cases among carriers exceeded 100. Associations that reached a significance threshold of $p < \frac{0.05}{6483 \times 67405} = 1.14 \times 10^{-10}$ are reported in Supplementary Data 8.

6.3 Detailed analysis of the *GLS* locus

We investigated the *GLS* 5' UTR CAG repeat locus in more detail in light of the associations that we identified with quantitative traits (Supplementary Data 7) as well the role of highly expanded *GLS* repeats in autosomal recessive glutaminase deficiency with impaired intellectual development and progressive ataxia [54].

In these follow-up analyses, we subdivided long *GLS* repeat alleles ($\geq$45 repeat units) into those that were highly expanded ($\sim$100+ repeats, based on the presence of $\geq$1 IRR pair) and those that were not, and we also examined SNPs and indels predicted to cause loss of function (pLoF). We identified carriers of *GLS* pLoF variants using the UKB WES genotype calls [28], restricting to variants annotated as pLoF by gnomAD v4.0.0 [55] with no QC flags (`lc_lof` or `lof_flag`). We additionally filtered two indel variants (`chr2:190931597:C:CA` and `chr2:190931597:CA:C`) that had much higher frequencies than expected in the UKB WES genotype calls.

We tested each of these three classes of *GLS* variants (highly expanded repeats, long but not highly expanded repeats, and pLoF variants) for association with the 57 heritable quantitative traits, and we also tested for association with glutamine levels measured in plasma (Fig. 5c). Whereas all three classes of variants associated with increased glutamine levels and decreased height with similar effect sizes, only highly expanded repeats associated with liver and renal biomarkers. Associations with highly expanded repeat status that reached Bonferroni significance ($p < 0.05/57$) are reported in Supplementary Table 8.

For each individual with a long *GLS* allele, we estimated *GLS* allele lengths as described previously (equation (3) from section 4.2.1), assuming any IRR pairs originated from the *GLS* locus. We restricted to individuals with EUR genetic ancestry, removing related individuals among the set of individuals with long *GLS* alleles. We then residualized standardized GGT, cystatin C, and glutamine for sex, age, and age squared. For quintiles of long *GLS* allele lengths, we computed the mean residualized phenotype and mean allele length. We also computed the mean residualized phenotype among individuals with no long *GLS* allele. Confidence intervals were computed using a t-test.

Given the large effects of highly expanded *GLS* repeat alleles on both liver and renal biomarkers, we tested highly expanded repeat status for association with ICD-10 categories and subcategories of liver diseases (K70–K77, Diseases of liver) and common kidney diseases (N17–N19, Acute kidney failure and chronic kidney disease) using the BinomiRare test. We obtained ICD-10 codes containing up to four characters from hospital episode statistics, cancer registry, and death registry data for UKB participants (accessed on 2022-10-26), from which we compiled 11 category-level ICD-10 disease phenotypes and 62 subcategory-level phenotypes, for a total of 73 liver and renal disease phenotypes.

Among the 11 association tests with top-level ICD-10 categories, three were significant (FDR-adjusted $p < 0.05$), and among the 62 association tests with ICD-10 subcategories, four were significant (FDR-adjusted $p < 0.05$); results are reported in Supplementary Table 9. The strongest association we observed was with the ICD-10 subcategory for stage 5 chronic kidney disease (N18.5). Upon closer examination of ICD-10 subcategory definitions within N18, we found that a separate code is used for stage 5 CKD requiring chronic dialysis (N18.6, or previously N18.0, for "end-stage renal disease"), such that combining these subcategories would provide a better phenotype for stage 5 CKD. We therefore focused on this phenotype (CKD stage 5, inclusive of end-stage renal disease) in our primary analyses.

6.4 Replication of *GLS* associations with liver and kidney disease in AoU (v7)

We identified carriers of highly expanded *GLS* repeats in the AoU (v7) WGS data set based on the presence of an anchored IRR at *GLS* and at least one IRR pair. We then attempted to replicate associations we observed in UKB between highly expanded repeat status and the following liver and kidney disease phenotypes:

- "Other diseases of liver" (K76), defined as an ICD-10 code of K76 or an ICD-9 code of 573.0, 572.3, 572.4, or 573.5 (corresponding to the ICD-10 subcategories of K76)
- Chronic kidney disease (N18), defined as the "Chronic kidney disease" concept in AoU (which aggregates both ICD-10 and ICD-9 codes)
- CKD stage 5, inclusive of end-stage renal disease, defined as an ICD-10 code of N18.5 or N18.6 or an ICD-9 code of 585.5 or 585.6.

We did not attempt to replicate the association with the ICD-10 top-level category K75 ("Other inflammatory liver diseases") as power was very limited due to low case prevalence in AoU, and similarly, we did not attempt to replicate associations with ICD-10 subcategories other than stage 5 CKD.

We performed association tests using logistic regression, adjusting for sex, age, age^2, and genetic ancestry, and restricting to an unrelated subset of individuals (removing one individual in each pair of relatives with kinship coefficient >0.1, retaining carriers of highly expanded *GLS* alleles when possible).

6.5 Assessment of X chromosome repeats for association with phenotypes

Our primary analysis pipeline excluded STRs on the X chromosome because the high-quality STR reference panel that we used to ascertain repeat expansions did not include STRs on the X chromosome [7]. To circumvent this difficulty, we implemented a reference-free approach to test potential X chromosome repeat expansions for associations with phenotypes. Specifically, we generated bin-level genotypes indicating, for each 1kb bin on chromosome X and for each possible repeat unit size (2bp, 3bp, 4bp, 5bp, 6bp), whether or not an individual had a highly repetitive read with the corresponding motif size whose mate aligned to the 1kb bin or one of its two flanking 1kb bins (building upon the reference-free approach described in Section 5.2.1). We identified 108,831 such bin+motif-size pairs on chromosome X for which at least 10 UKB participants were carriers of potential repeat expansions (i.e., had at least one highly repetitive read whose mate aligned within the 3kb window corresponding to the bin). We then conducted sex-stratified association analyses with the 6,483 binary disease phenotypes and 57 heritable quantitative traits, testing whether expansion status putatively encoded by any of these 108,831 measurements associated with any of

these phenotypes.

These analyses only identified disease associations involving two clusters of bin measurements (at chrX:154.341–154.343Mb and chrX:154.389–154.392Mb) that turned out to both reflect mis-mapping of reads derived from repeat expansions of the *C9orf72* CCCCGG repeat on chromosome 9. Most individuals genotyped to be "carriers" of these bin-level measurements had expanded *C9orf72* CCCCGG repeats (>80%), and the associated diseases were the same neurodegenerative and dementia-related diseases that associated with *C9orf72* expansions (Supplementary Data 8).

Similarly, the analyses of quantitative traits also identified only associations that appeared to reflect mis-mapping of reads derived from expanded autosomal STRs to loci on the X chromosome:

- associations of bins capturing mis-mapped *C9orf72* CCCCGG repeat expansions with reduced BMI and monocyte counts
- associations of bins capturing mis-mapped *CNBP* TCTG repeat expansions (actually located on chromosome 3, and pathogenic for myotonic dystrophy 2) with reduced creatinine
- associations of bins in the Xq telomere (likely capturing reads derived from telomere repeats on many chromosomes) with increased telomere length
- associations of bins in the Xp subtelomeric region (containing only a 26bp VNTR but not STR, and thus likely reflecting mis-mapping) with decreased neutrophil and white blood cell counts.

These results demonstrate the challenges of short-read-based analyses of STRs and the importance of using a reference panel for such analyses.

7 Supplementary Figures

a

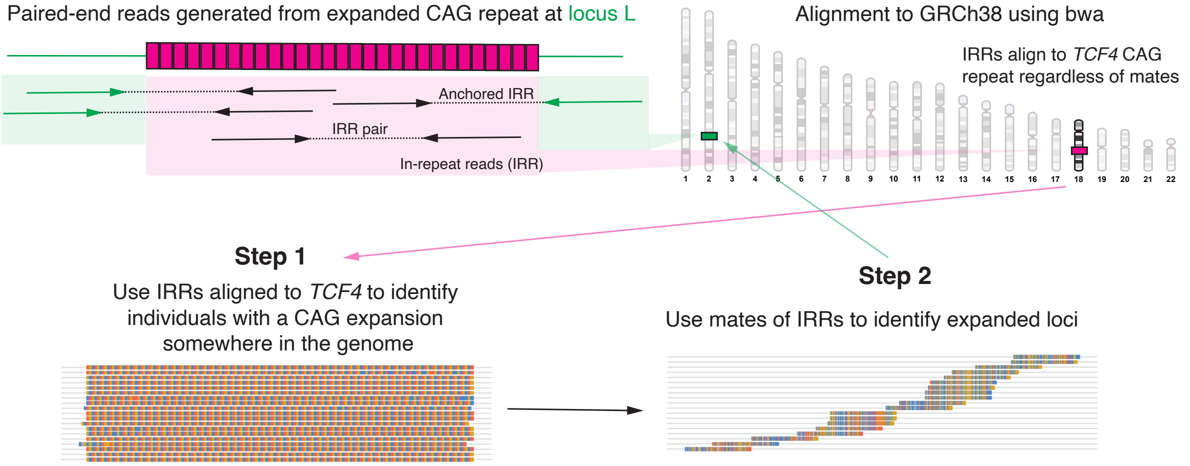

b

	Number of IRRs observed among 500 random WGS samples		
Occurrences of repeat motif in read SEQ	IRRs mapped to *TCF4*	IRRs mapped elsewhere with mates mapped to *CA10*	Other IRRs
45	84	38	9
46	94	20	4
47	115	7	5
48	132	11	4
49	170	10	7
50	643	5	0
45-50	1238	91	29

c

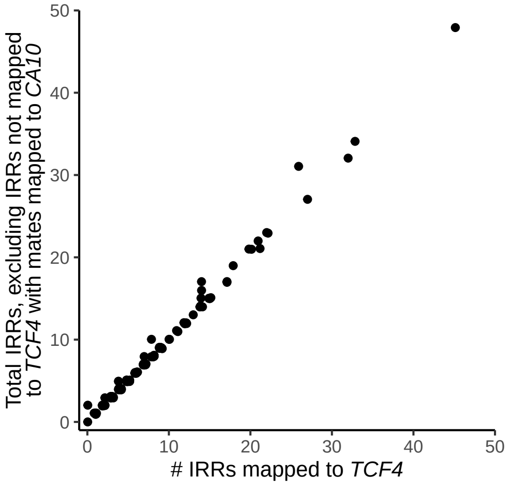

Supplementary Figure 1. Overview of approach to identify long CAG repeats from short-read WGS read alignments. a, Individuals with long CAG repeat alleles have WGS reads consisting entirely or almost entirely of CAG repeats (in-repeat reads, IRRs). The bwa aligner [5] maps most such reads to the CAG repeat in *TCF4* on chromosome 18 (which is the longest CAG repeat in the GRCh38 reference genome), such that searching for IRRs among reads aligned to *TCF4* reveals which individuals have long CAG repeats somewhere in their genome. By examining the mapping locations of uniquely-mapped mates of IRRs (“anchoring” these IRRs), we can determine where in the genome these repeat sequences originated. **b**, Mapping of IRRs found across 500 randomly selected WGS cram files (of which 164 had at least one IRR). Each IRR is classified as either (i) mapping to *TCF4*, (ii) not mapping to *TCF4* but with a mate that mapped to *CA10*, or (iii) not mapping to *TCF4* and with a mate that mapped to locus other than *CA10*. **c**, The number of IRRs per individual satisfying (i) or (iii) is plotted versus the number satisfying (i).

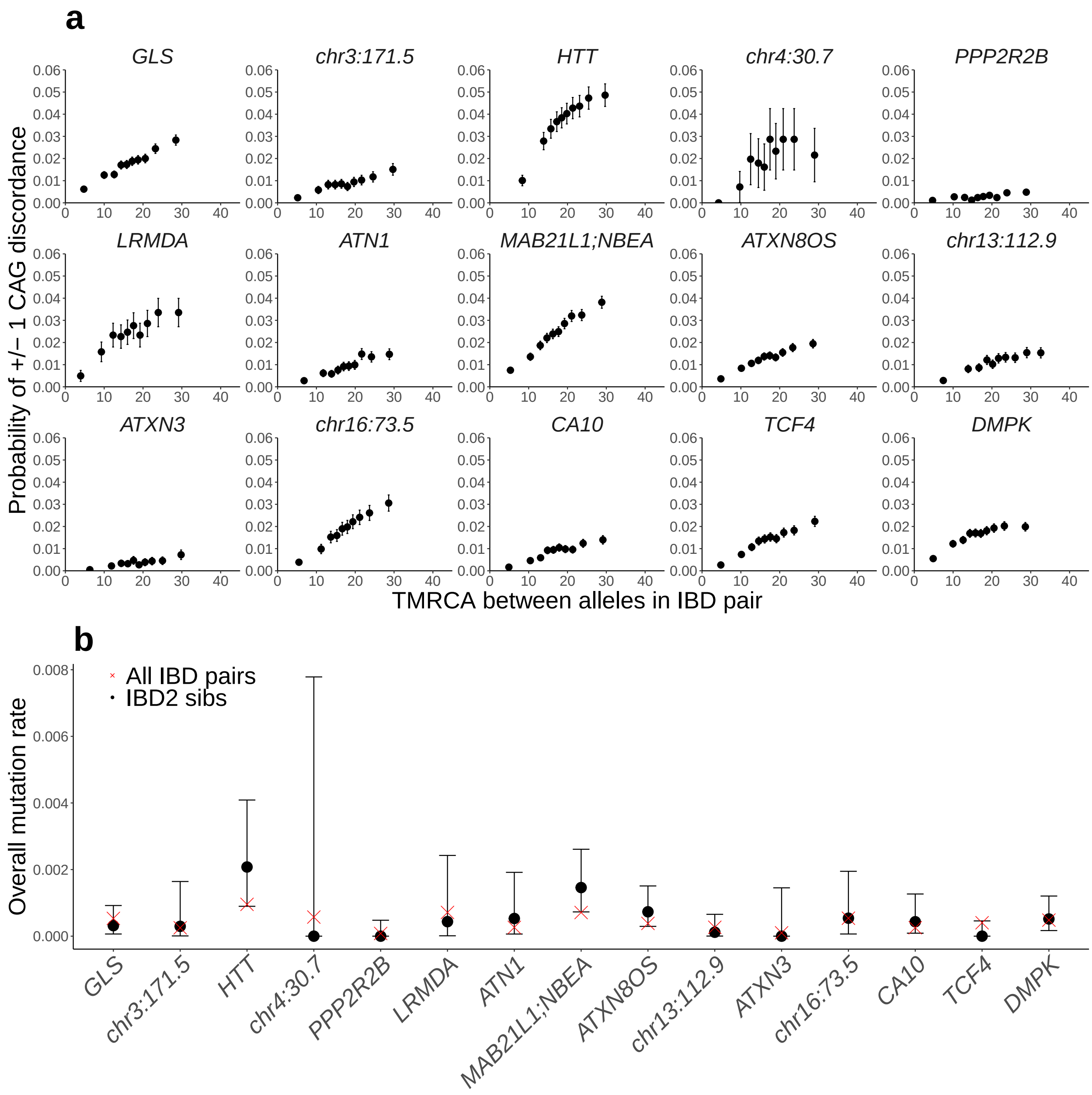

Supplementary Figure 2. Validation of germline mutation rates of CAG repeats. a, Discordance rate among alleles shared within IBD segments as a function of estimated TMRCA (binned into deciles). Discordance rates scale approximately linearly with TMRCA, as expected if discordances mostly reflect recent germline mutations and if TMRCA is accurately estimated. **b**, Rates of ±1 CAG mutations per transmission of each repeat directly estimated from IBD2 sibling pairs (black dots; error bars, 95% CIs) and estimated from all IBD pairs used in our analyses of allele-specific germline mutation rates (red crosses). Sample sizes available in Supplementary Table 3.

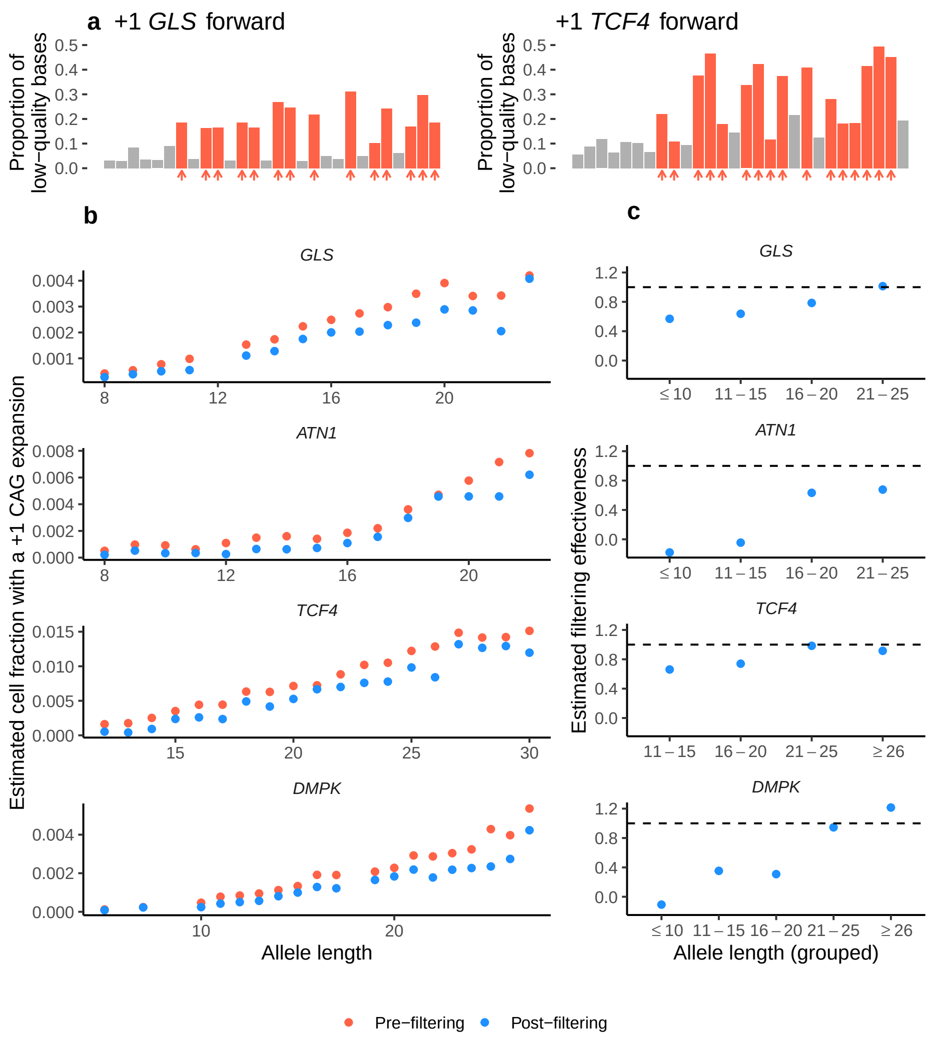

Supplementary Figure 3. Effect on somatic instability analysis of filtering aberrant reads with evidence of PCR stutter error. a, PCR stutter errors during bridge amplification generate "barcodes" of reduced-quality base calls at positions following the repeat at which the original sequence differs from the expanded/contracted sequence (see Fig. 1c). For *GLS* (left) and *TCF4* (right), proportions of low-quality bases among UKB WGS forward-strand reads supporting a +1 repeat unit expansion (some truly derived from somatic mutations, others generated by PCR error) are shown at the final six base positions of the repeat and positions thereafter. Bases at which PCR stutter error is predicted to reduce base quality are indicated with red arrows. **b**, Estimated fractions of blood cells in which AoU participants heterozygous for a repeat allele of a given length had experienced a +1 repeat unit expansion, computed either using all reads (pre-filtering) or applying our filtering approach. **c**, Estimated filtering effectiveness (i.e., proportion of reads passing filtration that were truly derived from somatically-expanded +1 repeat alleles) as a function of allele length. These estimates were obtained by regressing the fraction of cells estimated to have a +1 repeat unit expansion on age in AoU. The total number of haplotypes analyzed varied by locus (minimum n=143,297; maximum n=248,537).

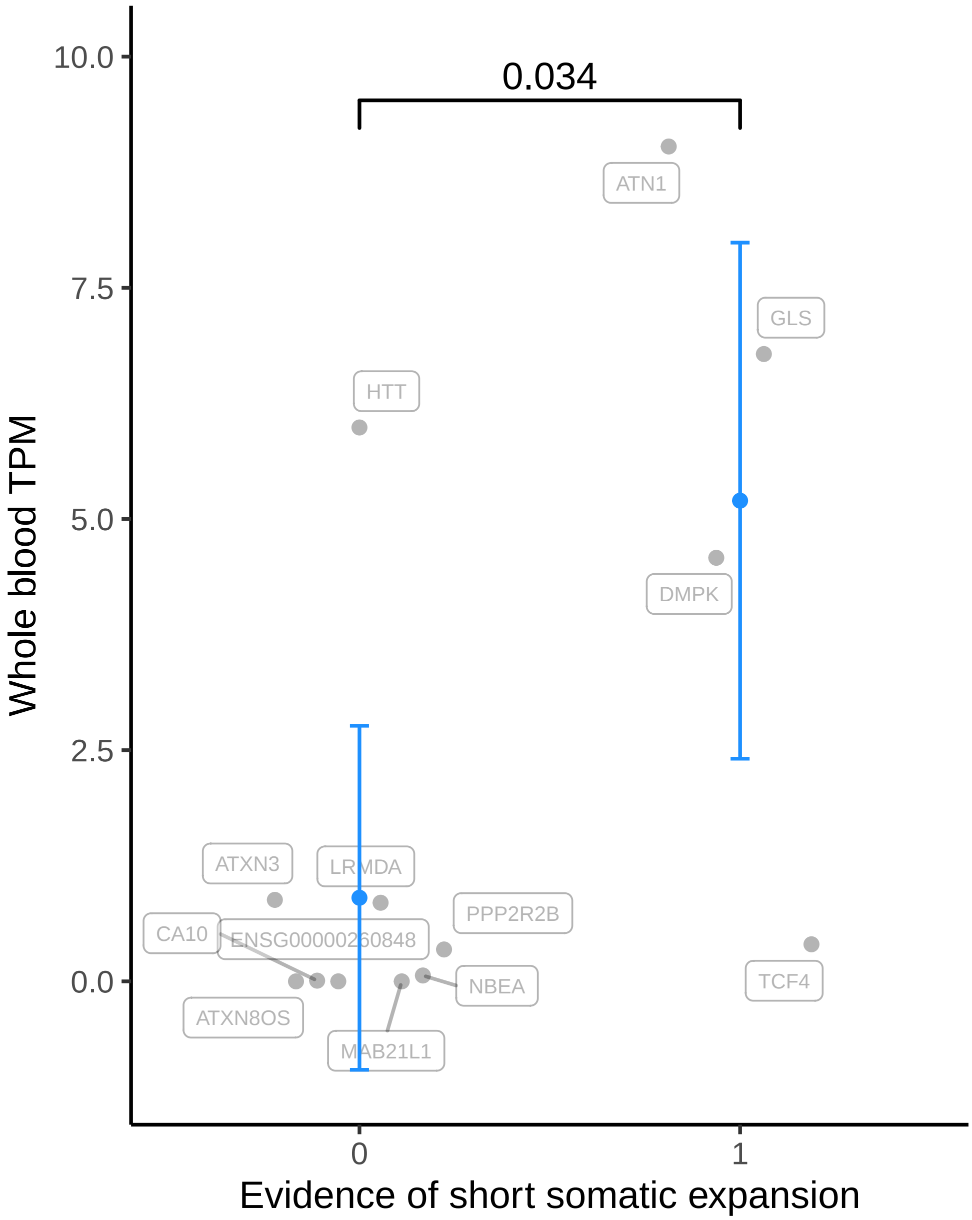

Supplementary Figure 4. Transcription levels in blood for genes for which repeat expansions are (or are not) expansion-prone in blood. For each of the 13 genes containing a repeat included in our analyses (Extended Data Fig. 1b), we obtained its median expression in blood (transcripts per million; TPM) from GTEx v8 [56]. We then classified genes based on whether or not we had identified evidence of somatic repeat instability in blood (Extended Data Fig. 3). Each group's mean transcription level (and 95% confidence interval) is shown in blue; p-value for the difference between the groups was computed using the Wilcoxon rank-sum test and shown above.

8 Supplementary Tables

Repeat region (GRCh38)	Genic context	Repeat composition	Flanking sequence Start	End
chr2:190880873-190880920	*GLS*	GCA_n	TAGCGCGCA	GCACCCGCA
chr3:171524288-171524320	Intergenic	CTG_n	AGAACACTG	CTGACTCAG
chr4:3074877-3074968	*HTT*	$CAG_n(CAACAG)_{1,2}$	TCCTTCCAG	CAGCCGCCA
chr4:30716894-30716931	Intergenic	AGC_n	CGCCAAAGC	AGCAGGCTC
chr5:146878728-146878759	*PPP2R2B*	GCT_n	CTCGCAGCT	GCTGCAGGA
chr6:16327634-16327724	*ATXN1*	$TGC_{n_1}(TGATGC)_{n_2}TGA(TGC)_{n_3}$	CTGAGGTGC	TGCTCAGCC
chr10:75973021-75973073	*LRMDA*	CAG_n	TAACAACAG	CAGCTACTC
chr11:93535787-93535815	*SRP14P2;SMCO4*	$CAG_nCAA_1CAG_2$	CCAAAGCAG	CAGCACAGT
chr12:6936717-6936773	*ATN1*	$(CAGCAA)_2(CAG)_n$	CACCACCAG	CAGCATCAC
chr13:35476296-35476355	*MAB21L1;NBEA*	CTG_n	GTTTCCCTG	CTGCTTTTC
chr13:67656824-67656870	lncRNA	AGC_n	AATTGGAGC	AGCAGGAAA
chr13:70139352-70139429	*ATXN8OS*	CTG_n	CTACTACTG	CTGCATTTT
chr13:112934556-112934581	Intergenic	TGC_n	AGGGGATGC	TGCTGGTCT
chr14:92071009-92071053	*ATXN3*	$(CTG)_n(TTGCTG)_{0,1}CTTTTG(CTG)_2$	GTCCCCCTG	CTGTCTGAA
chr16:73546663-73546741	lncRNA	$(TGC)_2TGT(TGC)_nTGTTGC$	TTTGGGTGC	TGCTTCTTT
chr17:51831667-51831732	*CA10*	AGC_n	AGAAAAAGC	AGCAGAAAA
chr18:55586154-55586228	*TCF4*	AGC_n	AGGAGGAGC	AGCATGAAA
chr19:45770205-45770264	*DMPK*	CAG_n	TCCCCCCAG	CAGCATTCC

Supplementary Table 1. Locations and sequence contexts of CAG repeat loci expanded to $\geq$45 repeat units in $\geq$5 UKB participants. Our analysis pipeline used anchored IRRs to approximately localize long repeats to their loci of origin, after which we obtained base-pair-resolution information about repeat locations from ref. [7]. The repeat length polymorphism we measured corresponds to the subscript n in the repeat composition column. The *ATXN1* repeat (chr6:16327634–16327724) contains two distinct length-polymorphic CAG sequences, so we dropped this repeat from downstream analysis along with two other repeats that failed other filters, leaving the 15 loci shown in Extended Data Fig. 1b. Repeat expansions in genes highlighted in red are known to be pathogenic. For *HTT* and *ATXN3*, repeat alleles contain low-frequency polymorphisms that flank the core repeat (as indicated in the repeat compositions in the table). The common repeat composition of *HTT* repeats is $CAG_n(CAACAG)_1$, and the common repeat composition of *ATXN3* repeats is $(CTG)_n(TTGCTG)_1CTTTTG(CTG)_2$, so we restricted analyses of germline mutation rates to these alleles (Extended Data Fig. 2). Allele frequencies of the low-frequency alleles $CAG_n(CAACAG)_2$ (*HTT*) and $(CTG)_nCTTTTG(CTG)_2$ (*ATXN3*) are shown in Extended Data Fig. 1b in blue.

Genic context	# CAG repeat loci	# expanded	OR (95% CI)	p-value
intergenic	328	3	0.5 (0.09-1.79)	0.43
transcript	831	15	1.99 (0.56-10.8)	0.43
exon	331	10	3.19 (1.12-9.39)	0.016
CDS	230	4	1.16 (0.27-3.73)	0.77
UTR	73	4	4.43 (1.03-14.61)	0.023

Supplementary Table 2. Enrichment of CAG repeats expanded to $\geq$45 repeat units in $\geq$5 UKB participants in various genic contexts. Odds ratios, confidence intervals, and p-values are from Fisher's exact test for association between genic context of a CAG repeat and whether or not the repeat was expanded in $\geq$5 UKB participants).

Locus	Number of confident heterozygotes: Allele length difference ≥ 1	≥ 3	≥ 5	Number of unique IBD-pairs: > 5 cM	> 5 cM & ANC	> 5 cM & ANC & $\lvert\Delta\rvert \leq 2$	Number of haplotypes: All	Unique	Average germline mutation rate
GLS	354484	268209	231303	213512	208368	200932	401864	286128	0.00053
chr3:171.5	176038	134842	114231	86067	84973	82782	165564	124339	0.00025
HTT	272762	148682	84159	78575	74645	68529	137058	106110	0.00095
chr4:30.7	171102	17462	12075	6035	5922	5579	11158	8322	0.00057
PPP2R2B	271392	215381	97461	151276	149628	147635	295270	217255	8.2×10^{-5}
LRMDA	304726	68799	45685	33003	32129	30430	60860	45753	0.00071
ATN1	253517	153282	126998	98445	96816	93953	187906	141110	0.00026
MAB21L1;NBEA	317113	256846	199996	207649	202156	192355	384710	276573	0.00071
ATXN8OS	361975	274016	136869	207196	203359	199431	398862	284668	0.00038
chr13:112.9	377876	258199	231065	112813	111666	108884	217768	172095	0.00027
ATXN3	121309	105116	85906	60990	60378	59418	118836	91514	0.0001
chr16:73.5	256239	155782	119419	93849	91050	85372	170744	127631	0.00054
CA10	234942	196668	171897	152276	149825	146025	292050	212087	0.00025
TCF4	275549	240444	147889	167199	164140	159998	319996	234563	0.0004
DMPK	363551	282071	259965	211754	206939	201754	403508	294221	0.00048

Supplementary Table 3. Average germline mutation rates of CAG repeats and sample sizes used in germline mutation rate analyses. The first set of columns provides the numbers of individuals heterozygous for two alleles with the indicated minimum allele length differences; we used $\geq$3 in our analyses. The second set of columns provides the numbers of longest IBD pairs satisfying progressive filters: $>$5 cM length with $>$0.5 cM on each flank; ancestral allele identified; jump size at most 2. The third pair of columns indicates the numbers of total and unique haplotypes involved in the final filtered set of longest IBD pairs. The final column provides the average per-generation 1-repeat-unit mutation rate based on all IBD pairs (dividing the total number of 1-repeat-unit discordances by twice the total TMRCA, adjusting expected TMRCAs for ascertainment of longest IBD matches).

		Meta-analysis			UKB (n=40,231)		AoU v7 (n=8,217)	
Variant (GRCh38)	Gene/locus	AF (%)	Beta (s.e.) s.d. units	P	Sign	P	Sign	P
1:67554471:T:C	*GADD45A*	96.91	0.11 (0.02)	2.9×10^{-8}	+	3.7×10^{-7}	+	0.026
5:80638411:G:T	*MSH3*	36.83	0.1 (0.0064)	2×10^{-52}	+	1.4×10^{-49}	+	3.4×10^{-5}
7:6027388:A:C	*PMS2*	20.23	0.04 (0.0078)	3×10^{-8}	+	3.4×10^{-8}	+	0.22
14:36541884:A:G	*SFTA3*, *NKX2-1*	7.17	0.07 (0.012)	3.4×10^{-8}	+	4.1×10^{-7}	+	0.028
14:54631346:A:G	*SAMD4A*	74.29	-0.04 (0.0071)	2.4×10^{-8}	-	6.6×10^{-7}	-	0.011
15:30902611:T:C	*FAN1*	49.15	-0.07 (0.0062)	8.5×10^{-29}	-	8.5×10^{-29}	-	0.019
17:30887878:C:G	*ATAD5*	26.73	0.05 (0.007)	4.9×10^{-12}	+	2.4×10^{-9}	+	0.00033

Supplementary Table 4. Genetic associations with somatic expansion of *TCF4* repeat alleles in blood. Allele frequencies, effect sizes (beta), and p-values are reported for lead variants from loci that reached genome-wide significance ($p < 5 \times 10^{-8}$) in GWAS meta-analysis of somatic instability of long TCF4 alleles in UKB and AoU v7 (restricting to long-allele carriers with age at least 40). Effect directions and p-values within each cohort (UKB and AoU) are provided.

				Somatic *HTT* CAG expansion in blood (ref. [39])		Somatic *TCF4* CAG expansion in blood (UKB+AoU)	
Locus	Tag variant	Modifier haplotype	MAF (%)	Beta	p-value	Beta	p-value
MSH2,MSH6	chr2:47491330:T:C	2ABEM1	1.4	-0.23	3.7×10^{-42}	-0.00	0.96
MSH2,MSH6	chr2:47978940:**T**:G	2ABEM2	37.0	0.03	1.3×10^{-9}	-0.02	0.0097
HTT	chr4:3074723:C:T	4ABEM1	3.4	0.13	4.8×10^{-30}	-0.02	0.35
MSH3	chr5:80632699:T:C	5ABEM1	24.4	0.04	7.2×10^{-19}	-0.08	6.9×10^{-25}
MSH3	chr5:80660180:T:G	5ABEM3	24.5	-0.04	4.6×10^{-21}	0.05	2.7×10^{-12}
MSH3	chr5:80790685:A:G	5ABEM2	0.4	-0.22	1.2×10^{-10}	0.01	0.79
PMS2	chr7:5978813:A:G	7ABEM3	4.4	0.05	8.4×10^{-8}	0.05	0.0018
PMS2	chr7:5986899:C:T	7ABEM1	1.8	0.10	9.4×10^{-11}	0.05	0.021
PMS2	chr7:6006003:T:C	7ABEM4	1.0	0.09	1.9×10^{-6}	0.08	0.0022
PMS2	chr7:6021277:**A**:G	7ABEM5	18.0	0.03	1.5×10^{-7}	0.02	0.058
PMS2	chr7:6063110:A:G	7ABEM2	6.4	0.05	3.4×10^{-8}	0.03	0.023
MLH3	chr14:75017975:**GTC**:G	14ABEM1	46.8	-0.03	4.1×10^{-10}	0.02	0.00043
MLH3	chr14:75017978:**T**:A	14ABEM1	46.8	-0.03	4.1×10^{-10}	0.02	0.00043
FAN1	chr15:30873651:**C**:T	15ABEM2	44.2	0.05	3.3×10^{-39}	0.06	3.9×10^{-23}
FAN1	chr15:30905792:C:T	15ABEM3	0.8	0.15	4.9×10^{-12}	0.16	1×10^{-5}
FAN1	chr15:30910758:G:A	15ABEM1	0.9	0.18	2.5×10^{-18}	0.20	6.6×10^{-11}
FAN1	chr15:30912434:C:T	15ABEM4	1.8	0.07	6.4×10^{-6}	0.10	1.6×10^{-5}
ATAD5, ADAP2,TEFM	chr17:30836573:T:A	17ABEM1	4.3	0.06	4.9×10^{-9}	0.07	1.8×10^{-5}
ATAD5, ADAP2,TEFM	chr17:30936682:C:T	17ABEM2	22.3	0.03	8.5×10^{-8}	0.04	2.2×10^{-7}

Supplementary Table 5. Effects of haplotypes influencing *HTT* repeat expansion in blood on *TCF4* repeat expansion in blood. For each tag variant previously associated with somatic expansion of the *HTT* repeat in blood DNA (Table S2 of ref. [39]), the effect size and p-value from our meta-analysis of *TCF4* somatic expansion in UKB+AoU are shown. The effect allele is the minor allele, which is the ALT allele for most variants; for variants for which the effect (minor) allele is the REF allele, it is typeset in bold and underlined. Effect sizes (betas) for somatic expansion of *HTT* are in units of the adjusted somatic expansion ratio (SER) defined in ref. [39]; for *TCF4*, units of betas are standard deviations of our *TCF4* somatic-expansion phenotype. Haplotype nomenclature at modifier loci was defined in ref. [39] using a scheme following ref. [35]: chromosome number, order of locus discovery on given chromosome (i.e., A, B, ...), ‘BE‘ for blood expansion, and a sequential number for each modifier at given locus (i.e., M1, M2, M3, ...).

				Age at onset		Age at SDMT30		Somatic *TCF4* expansion	
Variant	Gene	Modifier	MAF	β	P	β	P	β	P
2:190579929:A:C	*PMS1*	2AM1	20.63	-0.11	4.9×10^{-9}	-0.086	6.6×10^{-5}	-0.0008	0.91
2:190639862:C:T	*PMS1*	2AM2	1.28	0.3	3.7×10^{-6}	0.17	0.026	0.0081	0.81
3:37068079:T:C	*MLH1*	3AM1	31.41	0.11	3×10^{-11}	0.088	4.5×10^{-6}	-0.016	0.019
3:37121844:G:A	*MLH1*	3AM1	31.52	0.11	1.1×10^{-10}	0.09	2.5×10^{-6}	-0.017	0.014
4:2533199:G:A	*HTT*	CAA-loss	0.32	-1.2	4.8×10^{-9}	-2.1	1.8×10^{-9}		
4:2798279:C:T	*HTT*	CAA-loss	0.36	-1.2	4.9×10^{-9}	-2	3.1×10^{-10}		
4:2971698:A:G	*HTT*	CAACAG-dup	0.60	0.52	1.7×10^{-7}	0.49	4.2×10^{-5}	-0.11	0.18
5:79913275:G:A	*MSH3*	5AM1	25.44	-0.12	1×10^{-11}	-0.25	1.4×10^{-36}	-0.076	3.6×10^{-25}
5:79950781:**A**:G	*MSH3*	5AM1	25.36	-0.12	2.6×10^{-11}	-0.25	4.7×10^{-37}	-0.075	1.8×10^{-24}
5:80086504:A:G	*MSH3*	5AM2	0.32	0.74	5.7×10^{-9}	0.59	2.9×10^{-5}	0.012	0.79
5:79961856:AT:A	*MSH3*	5AM3	27.78	0.078	5.6×10^{-6}	0.19	2×10^{-22}	0.097	4.2×10^{-44}
5:79990883:T:G	*MSH3*	5AM3	33.14	0.084	2.6×10^{-7}	0.17	1.4×10^{-19}	0.075	3.7×10^{-30}
5:145886836:G:A	*TCERG1*	5BM1	2.71	0.32	3.3×10^{-12}	0.21	3.8×10^{-5}	-0.014	0.47
7:6022626:C:T	*PMS2*	7AM1	14.67	0.12	6.4×10^{-9}	0.13	1.3×10^{-7}	-0.02	0.04
7:6056484:G:C	*PMS2*	7AM2	18.41	-0.099	2.4×10^{-7}	-0.088	6.7×10^{-5}	-0.0046	0.55
7:6041836:T:A	*PMS2*	7AM3	41.42	0.075	8.3×10^{-7}	0.12	4.7×10^{-12}	-0.03	1.5×10^{-6}
7:6026530:C:T	*PMS2*	7AM4	2.14	-0.17	0.00064	-0.27	5.5×10^{-6}	0.049	0.021
7:56241504:C:T	*?*	7BM1	2.94	0.26	7.3×10^{-9}	0.098	0.06	0.021	0.22
8:103213640:G:T	*RRM2B*	8AM1	7.91	-0.16	2.1×10^{-9}	-0.09	0.0042	-0.013	0.33
11:96079307:C:A	*CCDC82*	11AM1	19.53	0.096	4.2×10^{-7}	0.084	9.2×10^{-5}	-0.0004	0.94
15:31202961:G:A	*FAN1*	15AM1	1.24	-0.69	3.8×10^{-28}	-0.7	1.2×10^{-20}	0.2	6.6×10^{-11}
15:31247852:G:T	*FAN1*	15AM1	1.15	-0.7	1.3×10^{-26}	-0.75	4.1×10^{-21}	0.23	5.2×10^{-13}
15:31241346:G:A	*FAN1*	15AM2	27.81	0.19	7.2×10^{-28}	0.19	1.3×10^{-21}	0.053	9.7×10^{-15}
15:31197995:C:T	*FAN1*	15AM3	0.82	-0.59	1.8×10^{-13}	-0.49	6.5×10^{-8}	0.16	1×10^{-5}
15:31204637:C:T	*FAN1*	15AM5	1.88	-0.33	4.5×10^{-10}	-0.18	0.004	0.096	1.6×10^{-5}
19:48622545:A:G	*LIG1*	19AM1	17.19	0.12	1.2×10^{-9}	0.078	0.00071	0.018	0.26
19:48687051:ACC:A	*LIG1*	19AM1	12.60	0.11	6.2×10^{-7}	0.11	9×10^{-6}	-0.012	0.2
19:48643050:CCT:C	*LIG1*	19AM2	36.87	-0.083	1.2×10^{-7}	-0.057	0.0016	-0.0073	0.29
19:48620943:C:A	*LIG1*	19AM3	0.17	1.2	5.9×10^{-11}	0.89	1.2×10^{-5}	-0.17	0.022

Supplementary Table 6. Effects of haplotypes influencing age-at-onset and age-at-SDMT30 for Huntington's disease on *TCF4* repeat expansion in blood. For each tag variant previously associated with age-at-onset (z-score) or age-at-SDMT30 (z-score) for Huntington's disease (Table S1 of ref. [48]), the effect size and p-value from our meta-analysis of *TCF4* somatic expansion in UKB+AoU are shown. The effect allele is the ALT allele for all but one variant; for the 5AM1 variant for which the effect allele is the REF allele, it is typeset in bold and underlined. Haplotype nomenclature at modifier loci was defined in ref. [35] and indicates chromosome number, order of locus discovery on given chromosome (i.e., A, B, ...) and a sequential number for each modifier at given locus (i.e., M1, M2, M3, ...).

a

Gene	Burden mask	AF	β	s.e.	p
		chr19:14.8Mb AAAG long alleles			
MLH1	missense8.0.001	0.0018	-0.19	0.02	4.2×10^{-15}
MLH3	missense8.0.001	0.0014	-0.24	0.03	4.4×10^{-17}
MSH2	all.transcripts.missense6.0.01	0.0048	-0.19	0.02	8.6×10^{-38}
MSH3	all.transcripts.CNV.missense6.0.01	0.0075	-0.32	0.01	2.6×10^{-157}
MSH6	all.transcripts.CNV.missense7.0.001	0.004	-0.12	0.02	1.1×10^{-12}
NEIL2	all.transcripts.CNV.missense6.0.001	0.0011	0.14	0.03	1.7×10^{-6}
PMS1	all.transcripts.CNV.missense7.0.01	0.0033	-0.30	0.02	1.8×10^{-58}
PMS2	all.transcripts.CNV.missense8.0.001	0.002	0.25	0.02	3.9×10^{-28}
XPC	all.transcripts.CNV.missense6.0.0001	0.0016	0.19	0.03	6.3×10^{-14}
		chr2:232.4Mb AAAG mid-length alleles			
MSH2	all.transcripts.missense6.0.01	0.0047	-0.20	0.02	1.9×10^{-20}
MSH3	all.transcripts.CNV.missense7.0.01	0.0067	-0.35	0.02	1.8×10^{-79}
MSH6	all.transcripts.CNV.missense7.0.001	0.0041	-0.13	0.02	1.3×10^{-8}
NEIL2	all.transcripts.missense8.singleton	9.2×10^{-6}	1.70	0.50	0.00055
PMS1	missense7.0.01	0.0032	-0.30	0.03	6.3×10^{-30}
PMS2	all.transcripts.CNV.missense7.0.001	0.0028	0.21	0.03	2.8×10^{-14}

b

	chr19:14.8Mb AAAG long alleles				chr2:232.4Mb AAAG mid-length alleles			
Gene	AF	β	s.e.	p	AF	β	s.e.	p
MLH3	8.8×10^{-5}	-0.28	0.11	0.013	9×10^{-5}	-0.12	0.16	0.44
MSH2	8.9×10^{-5}	-0.30	0.11	0.0079	7.6×10^{-5}	-0.26	0.17	0.074
MSH3	0.00063	-0.57	0.04	2.6×10^{-43}	0.00062	-0.53	0.06	1.6×10^{-18}
MSH6	0.00059	-0.16	0.04	0.00017	0.0006	-0.29	0.06	4.2×10^{-6}
NEIL2	0.00023	0.22	0.07	0.0015	0.00021	0.15	0.10	0.17
PMS1	0.0005	-0.39	0.05	1.5×10^{-16}	0.00048	-0.25	0.07	0.00023
PMS2	0.00016	0.44	0.08	6.1×10^{-8}	0.00014	0.36	0.12	0.004
XPC	0.00038	0.32	0.05	4.8×10^{-10}	0.00036	-0.12	0.08	0.16

Supplementary Table 7. Associations of rare coding variation with AAAG somatic repeat expansion in gene-level burden tests. **a**, Genes for which at least one burden mask (from among up to 64 masks considered per gene [43]) associated with optimized AAAG somatic-expansion phenotypes ($p < 5 \times 10^{-8}$, correcting for $\sim$1 million total tests). For each gene, data are shown for the burden mask that generated the most significant association. Data are also shown for *NEIL2*, which did not reach the Bonferroni significance threshold but was of interest because of the significant common-variant signal at *NEIL2*. Burden mask names indicate whether they used all transcripts ("all.transcripts") or only the MANE select transcript [44]; whether they included CNVs [49] ("CNV"); whether they included missense variants, and if so, the PrimateAI-3D score threshold [42] (>0.6, 0.7, 0.8); and the maximum allele frequency allowed. **b**, Effect size estimates for pLoF variants (using the LoF.CNV.0.0001 mask) for the genes in panel **a**, excluding *MLH3* due to low AF (5×10^{-5}). Effect sizes are in units of standard deviations.

Category	Quantitative trait	*GLS* pLoF SNP/indel		Long but not highly expanded *GLS*		Highly expanded *GLS*	
		β (s.e.)	p-value	β (s.e.)	p-value	β (s.e.)	p-value
Anthropometric	Height	-0.29 (0.098)	0.0029	-0.44 (0.16)	0.0068	-0.47 (0.1)	6.3×10^{-6}
Blood	Lymphocyte Ct.	-0.14 (0.099)	0.15	-0.31 (0.17)	0.065	-0.51 (0.11)	2.4×10^{-6}
Blood	Platelet Distr. Width	0.021 (0.098)	0.83	-0.05 (0.17)	0.76	0.46 (0.11)	2×10^{-5}
Blood	Platelet Ct.	-0.032 (0.099)	0.75	-0.23 (0.17)	0.17	-0.38 (0.11)	0.00042
Bone and joint	Alkaline Phosphatase	0.058 (0.1)	0.56	0.18 (0.17)	0.29	0.71 (0.11)	1.2×10^{-10}
Bone and joint	Calcium	-0.046 (0.1)	0.65	0.14 (0.18)	0.45	0.56 (0.11)	6.3×10^{-7}
Bone and joint	Vitamin D	0.017 (0.1)	0.87	-0.077 (0.17)	0.66	-0.39 (0.11)	0.00052
Cancer	IGF1	-0.14 (0.1)	0.15	0.15 (0.17)	0.38	-0.38 (0.11)	0.0006
Liver	GGT	-0.083 (0.1)	0.41	0.15 (0.17)	0.37	0.96 (0.11)	3.5×10^{-18}
Liver	AST	-0.0071 (0.1)	0.94	0.26 (0.17)	0.13	0.5 (0.11)	7.5×10^{-6}
Liver	Albumin	-0.085 (0.1)	0.41	0.43 (0.18)	0.019	-0.41 (0.11)	0.00022
Renal	Cystatin C	0.027 (0.1)	0.79	0.02 (0.17)	0.91	0.59 (0.11)	7.8×10^{-8}
Renal	Phosphate	0.058 (0.1)	0.57	-0.01 (0.18)	0.95	-0.45 (0.11)	7.7×10^{-5}
	Glutamine	0.44 (0.13)	0.001	0.71 (0.22)	0.0011	0.78 (0.14)	1.9×10^{-8}

Supplementary Table 8. Associations of highly expanded *GLS* repeats with heritable quantitative traits. Bonferroni-significant associations ($p < 0.05/57$, adjusting for 57 traits tested) for highly expanded *GLS* repeat status (i.e., $\geq$1 IRR pair) are shown. Effect sizes and p-values for association tests of less-expanded *GLS* repeat status (i.e., anchored IRRs at *GLS*, but no IRR pairs) and *GLS* SNP/indel pLoF status are also provided. Effect sizes and p-values are from a joint model of pLoF status and expansion status (including both highly expanded and less-expanded repeat status as independent regressors), adjusting for sex, age, and age squared. Association results are also shown for glutamine. GGT, gamma-glutamyl transferase; AST, aspartate aminotransferase.

UK Biobank				
Phenotype		OR	95% CI	p-value
K75	Other inflammatory liver diseases	9.8	2.0-47.1	0.0043
K75.4	Autoimmune hepatitis	34.9	3.8-319.4	0.0017
K76	Other diseases of liver	3.6	1.4-8.8	0.0061
K76.8	Other specified diseases of liver	8.2	2.2-31.2	0.0019
N18	Chronic kidney disease (CKD)	4.5	2.3-9.0	1.6×10^{-5}
N18.5	Chronic kidney disease, stage 5	23.7	6.2-90.5	3.5×10^{-6}
N18.9	Chronic kidney disease, unspecified	5.3	2.2-12.8	0.00023
N18.0 or N18.5	CKD stage 5, inclusive of end-stage renal disease	20.6	5.5-76.8	7.0×10^{-6}
All of Us (v7)				
Other diseases of liver		2.3	0.8-6.7	0.12
Chronic kidney disease (CKD)		4.8	1.9-12.3	1.2×10^{-3}
CKD stage 5, inclusive of end-stage renal disease		10.1	3.0-34.2	1.9×10^{-4}
Meta-analysis				
Other diseases of liver		3.0	1.5-5.9	2.0×10^{-3}
Chronic kidney disease (CKD)		4.6	2.6-8.1	7.0×10^{-8}
CKD stage 5, inclusive of end-stage renal disease		14.0	5.7-34.3	7.2×10^{-9}

Supplementary Table 9. Associations of highly expanded *GLS* repeats with liver and kidney diseases. Associations that reached FDR<0.05 with top-level ICD-10 categories (3 significant out of 11 categories tested, K70–K77 and N17–N19) and associations that reached FDR<0.05 with ICD-10 subcategories (4 significant out of 62 subcategories tested) are listed. Upon closer examination of ICD-10 subcategory definitions within N18, we determined that combining N18.5 (chronic kidney disease, stage 5) and N18.0 (end-stage renal disease) would be a better choice for a CKD stage 5 phenotype, so we ran an association test for this combined phenotype in UKB and took this phenotype forward (along with two category-level phenotypes) for replication in AoU. Finally, we meta-analyzed the UKB and AoU associations (log odds ratios) using a linear random-effects model (DerSimonian-Laird estimator) using the rma.uni() function within the metafor package in R.

References

1. Bycroft, C. *et al.* The UK Biobank resource with deep phenotyping and genomic data. *Nature* **562**, 203–209 (2018).

2. The UK Biobank Whole-Genome Sequencing Consortium. Whole-genome sequencing of 490,640 UK Biobank participants. *Nature* **645** (2025).

3. The All of Us Research Program Investigators. The "All of Us" Research Program. *New England Journal of Medicine* **381** (2019).

4. The All of Us Research Program Genomics Investigators. Genomic data in the All of Us Research Program. *Nature* (2024).

5. Li, H. & Durbin, R. Fast and accurate short read alignment with Burrows–Wheeler transform. *Bioinformatics* **25**, 1754–1760 (2009).

6. Behera, S. *et al.* Comprehensive genome analysis and variant detection at scale using DRAGEN. *Nature Biotechnology* (2025).

7. Ziaei Jam, H., Li, Y., DeVito, R., Mousavi, N. *et al.* A deep population reference panel of tandem repeat variation. *Nature Communications* (2023).

8. Byrska-Bishop, M., Evani, U. S., Zhao, X., Basile, A. O. *et al.* High-coverage whole-genome sequencing of the expanded 1000 Genomes Project cohort including 602 trios. *Cell* **185**, 3426–3440 (2022).

9. Hujoel, M. L. *et al.* Influences of rare copy-number variation on human complex traits. *Cell* **185**, 4233–4248 (2022).

10. Mitra, I. *et al.* Patterns of de novo tandem repeat mutations and their role in autism. *Nature* **589**, 246–250 (2021).

11. Kristmundsdottir, S. *et al.* Sequence variants affecting the genome-wide rate of germline microsatellite mutations. *Nature Communications* **14**, 3855 (2023).

12. Campbell, C. D., Chong, J. X., Malig, M. *et al.* Estimating the human mutation rate using autozygosity in a founder population. *Nature Genetics* (2012).

13. Palamara, P. F., Francioli, L. C., Wilton, P. R., Genovese, G. *et al.* Leveraging Distant Relatedness to Quantify Human Mutation and Gene-Conversion Rates. *The American Journal of Human Genetics* (2015).

14. Narasimhan, V. M., Rahbari, R., Scally, A. *et al.* Estimating the human mutation rate from autozygous segments reveals population differences in human mutational processes. *Nature Communications* (2017).

15. Tian, X., Browning, B. L. & Browning, S. R. Estimating the Genome-wide Mutation Rate with Three-Way Identity by Descent. *The American Journal of Human Genetics* (2019).

16. Tian, X., Cai, R. & Browning, S. R. Estimating the genome-wide mutation rate from thousands of unrelated individuals. *The American Journal of Human Genetics* (2021).

17. Palamara, P. F. *Population Genetics of Identity By Descent*. Ph.D. thesis, Columbia University (2014).

18. Cai, R., Browning, B. L. & Browning, S. R. Identity-by-descent-based estimation of the X chromosome effective population size with application to sex-specific demographic history. *G3* (2023).

19. R Core Team. *R: A Language and Environment for Statistical Computing*. R Foundation for Statistical Computing, Vienna, Austria (2022).

20. Shinde, D., Lai, Y., Sun, F. & Arnheim, N. *Taq* DNA polymerase slippage mutation rates measured by PCR and quasi-likelihood analysis: (CA/GT)n and (A/T)n microsatellites. *Nucleic Acids Research* **31**, 974–980 (2003).

21. Raz, O. *et al.* Short tandem repeat stutter model inferred from direct measurement of *in vitro* stutter noise. *Nucleic Acids Research* **47**, 2436–2445 (2019).

22. Sehgal, A., Ziaei Jam, H., Shen, A. & Gymrek, M. Genome-wide detection of somatic mosaicism at short tandem repeats. *Bioinformatics* **40** (2024).

23. Goodwin, S., McPherson, J. D. & McCombie, W. R. Coming of age: ten years of next-generation sequencing technologies. *Nature Reviews Genetics* **17** (2016).

24. Handsaker, R. E. *et al.* Large multiallelic copy number variations in humans. *Nature Genetics* **47**, 296–303 (2015).

25. Loh, P.-R., Genovese, G. & McCarroll, S. A. Monogenic and polygenic inheritance become instruments for clonal selection. *Nature* **584**, 136–141 (2020).

26. Hofmeister, R. J., Ribeiro, D. M., Rubinacci, S. & Delaneau, O. Accurate rare variant phasing of whole- genome and whole-exome sequencing data in the UK Biobank. *Nature Genetics* **55** (2023).

27. Mukamel, R. E. *et al.* Protein-coding repeat polymorphisms strongly shape diverse human phenotypes. *Science* **373**, 1499–1505 (2021).

28. Backman, J. D. *et al.* Exome sequencing and analysis of 454,787 UK Biobank participants. *Nature* **599**, 628–634 (2021).

29. Hujoel, M. L., Handsaker, R. E. *et al.* Protein-altering variants at copy number-variable regions influence diverse human phenotypes. *Nature Genetics* (2024).

30. Taliun, D., Harris, D., Kessler, M. *et al.* Sequencing of 53,831 diverse genomes from the NHLBI TOPMed Program. *Nature* (2021).

31. Loh, P.-R. *et al.* Efficient Bayesian mixed model analysis increases association power in large cohorts. *Nature Genetics* **47**, 284–290 (2015).

32. Loh, P.-R., Kichaev, G., Gazal, S., Schoech, A. P. & Price, A. L. Mixed-model association for biobank-scale datasets. *Nature Genetics* **50**, 906–908 (2018).

33. Willer, C. J., Li, Y. & Abecasis, G. R. METAL: fast and efficient meta-analysis of genomewide association scans. *Bioinformatics* (2010).

34. McLaren, W. *et al.* The Ensembl Variant Effect Predictor. *Genome Biology* **17**, 122 (2016).

35. Genetic Modifiers of Huntington's Disease (GeM-HD) consortium. CAG Repeat Not Polyglutamine Length Determines Timing of Huntington's Disease Onset. *Cell* (2019).

36. Dolzhenko, E. *et al.* ExpansionHunter Denovo: a computational method for locating known and novel repeat expansions in short-read sequencing data. *Genome Biology* **21**, 102 (2020).

37. Bonfield, J. K. *et al.* HTSlib: C library for reading/writing high-throughput sequencing data. *GigaScience* **10** (2021).

38. Halman, A., Dolzhenko, E. & Oshlack, A. STRipy: A graphical application for enhanced genotyping of pathogenic short tandem repeats in sequencing data. *Human Mutation* **43**, 859–868 (2022).

39. Genetic Modifiers of Huntington's Disease (GeM-HD) consortium. Genetic modifiers of somatic expansion and clinical phenotypes in Huntington's disease highlight shared and tissue-specific effects. *Nature Genetics* (2025).

40. Loh, P.-R. *et al.* Contrasting genetic architectures of schizophrenia and other complex diseases using fast variance components analysis. *Nature Genetics* **47**, 1385–1392 (2015).

41. Logsdon, G. A. *et al.* Complex genetic variation in nearly complete human genomes. *Nature* 1–12 (2025).

42. Gao, H. *et al.* The landscape of tolerated genetic variation in humans and primates. *Science* **380**, eabn8153 (2023).

43. Tang, D., Kamitaki, N., Mukamel, R. E., Rubinacci, S. & Loh, P.-R. Patterns and drivers of 43,617 mosaic chromosomal alterations in blood. *medRxiv* (2025).

44. Morales, J. *et al.* A joint NCBI and EMBL-EBI transcript set for clinical genomics and research. *Nature* **604**, 310–315 (2022).

45. Zheng, S. C. *et al.* A novel cell-type deconvolution algorithm reveals substantial contamination by immune cells in saliva, buccal and cervix. *Epigenomics* **10**, 925–940 (2018).

46. Soyfer, E. M. *et al.* Saliva as a feasible alternative to blood for interrogation of somatic hematopoietic variants. *Blood Neoplasia* **1**, 100012 (2024).

47. Wang, G., Sarkar, A., Carbonetto, P. & Stephens, M. A simple new approach to variable selection in regression, with application to genetic fine mapping. *Journal of the Royal Statistical Society: Series B (Statistical Methodology)* **82**, 1273–1300 (2020).

48. Lee, J.-M., Huang, Y., Orth, M. *et al.* Genetic modifiers of Huntington disease differentially influence motor and cognitive domains. *The American Journal of Human Genetics* (2022).

49. Hujoel, M. L. *et al.* Protein-altering variants at copy number-variable regions influence diverse human phenotypes. *Nature Genetics* (2024).

50. Barton, A. R., Hujoel, M. L., Mukamel, R. E., Sherman, M. A. & Loh, P.-R. A spectrum of recessiveness among Mendelian disease variants in UK Biobank. *American Journal of Human Genetics* **19**, 1298–1307 (2022).

51. Jadhav, B. *et al.* A phenome-wide association study of methylated GC-rich repeats identifies a GCC repeat expansion in *AFF3* associated with intellectual disability. *Nature Genetics* **56**, 2322–2332 (2024).

52. Manigbas, C. A. *et al.* A phenome-wide association study of tandem repeat variation in 168,554 individuals from the UK Biobank. *Nature Communications* **15**, 10521 (2024).

53. Sofer, T. BinomiRare: a robust test of the association of a rare variant with a disease for pooled analysis and meta-analysis, with application to the HCHS/SOL. *Genetic Epidemiology* **41**, 388–395 (2017).

54. van Kuilenburg, A. *et al.* Glutaminase Deficiency Caused by Short Tandem Repeat Expansion in GLS. *The New England Journal of Medicine* (2019).

55. Chen, S., Francioli, L. C. *et al.* A genomic mutational constraint map using variation in 76,156 human genomes. *Nature* (2024).

56. Aguet, F., Barbeira, A. N., Bonazzola, R., Brown, A. *et al.* The GTEx Consortium atlas of genetic regulatory effects across human tissues. *Science* **369**, 1318–1330 (2020).
