# Supplementary material for: Insights into DNA repeat expansions among 900,000 biobank participants
Source: Nature. 2026 Jan 7;650(8103):920–9. doi: 10.1038/s41586-025-09886-z (PMC12935551; doi:10.1038/s41586-025-09886-z)
Supplement: Supplementary file 2 — Reporting Summary [file 41586_2025_9886_MOESM2_ESM.pdf]

nature portfolio

Corresponding author(s): Margaux L.A. Hujoel

Last updated by author(s): October 29, 2025

Reporting Summary

Nature Portfolio wishes to improve the reproducibility of the work that we publish. This form provides structure for consistency and transparency in reporting. For further information on Nature Portfolio policies, see our Editorial Policies and the Editorial Policy Checklist.

Statistics

For all statistical analyses, confirm that the following items are present in the figure legend, table legend, main text, or Methods section.

n/a	Confirmed	
☐	☒	The exact sample size (*n*) for each experimental group/condition, given as a discrete number and unit of measurement
☐	☒	A statement on whether measurements were taken from distinct samples or whether the same sample was measured repeatedly
☐	☒	The statistical test(s) used AND whether they are one- or two-sided<br>*Only common tests should be described solely by name; describe more complex techniques in the Methods section.*
☐	☒	A description of all covariates tested
☐	☒	A description of any assumptions or corrections, such as tests of normality and adjustment for multiple comparisons
☐	☒	A full description of the statistical parameters including central tendency (e.g. means) or other basic estimates (e.g. regression coefficient) AND variation (e.g. standard deviation) or associated estimates of uncertainty (e.g. confidence intervals)
☐	☒	For null hypothesis testing, the test statistic (e.g. *F*, *t*, *r*) with confidence intervals, effect sizes, degrees of freedom and *P* value noted<br>*Give P values as exact values whenever suitable.*
☒	☐	For Bayesian analysis, information on the choice of priors and Markov chain Monte Carlo settings
☒	☐	For hierarchical and complex designs, identification of the appropriate level for tests and full reporting of outcomes
☐	☒	Estimates of effect sizes (e.g. Cohen's *d*, Pearson's *r*), indicating how they were calculated

Our web collection on statistics for biologists contains articles on many of the points above.

Software and code

Policy information about availability of computer code

Data collection	None.
Data analysis	Code has been provided on GitHub (https://github.com/mhujoel/STRs and https://github.com/poruloh/extractLongSTRs) and has been deposited on Zenodo (10.5281/zenodo.17419996). We additionally used the following open-source software packages: samtools 1.15.1; BOLT-LMM 2.4.2; R 3.6.3; susieR 0.12.35; SHAPEIT5 v5.1.1; METAL 2020-05-05.

For manuscripts utilizing custom algorithms or software that are central to the research but not yet described in published literature, software must be made available to editors and reviewers. We strongly encourage code deposition in a community repository (e.g. GitHub). See the Nature Portfolio guidelines for submitting code & software for further information.

Data

Policy information about availability of data

All manuscripts must include a data availability statement. This statement should provide the following information, where applicable:

- Accession codes, unique identifiers, or web links for publicly available datasets
- A description of any restrictions on data availability
- For clinical datasets or third party data, please ensure that the statement adheres to our policy

Summary association statistics for GWAS of somatic-expansion phenotypes are available at the NHGRI-EBI GWAS Catalog (GCST90704615 to GCST90704642). Summary statistics for association tests of repeat expansions with quantitative traits and diseases are available at https://data.broadinstitute.org/lohlab/

UKB_STR_expansion_sumstats/ and Zenodo (10.5281/zenodo.1741999663). Access to the following data resources used in this study is obtained by application: UK Biobank (http://www.ukbiobank.ac.uk/), All of Us (https://allofus.nih.gov/). We additionally used the following data resources: the 1000 Genomes+H3Africa STR reference panel generated by Ziaei Jam et al. (https://github.com/gymrek-lab/EnsembleTR), the STRipy database (https://stripy.org), and the GTEx eQTL/sQTL browser (https://gtexportal.org).

Research involving human participants, their data, or biological material

Policy information about studies with human participants or human data. See also policy information about sex, gender (identity/presentation), and sexual orientation and race, ethnicity and racism.

Reporting on sex and gender	We included sex as a covariate in analyses.
Reporting on race, ethnicity, or other socially relevant groupings	We used the inferred genetic ancestry of each participant (uses their coordinates along the top 20 genetic principal components; see Methods) to define the primary analysis set.
Population characteristics	Prospective cohort study (~500,000 individuals from across the United Kingdom); individuals were between 40 and 69 years old at recruitment (Sudlow et al. 2015 PLOS Medicine).
Recruitment	Recruitment into UK Biobank has been described previously (Sudlow et al. 2015 PLOS Medicine).
Ethics oversight	This research complies with all relevant ethical regulations. The study protocol was determined to be not human subjects research by the Broad Institute Office of Research Subject Protection and the Partners HealthCare Human Research Committee (as all data analyzed were previously collected and de-identified).

Note that full information on the approval of the study protocol must also be provided in the manuscript.

Field-specific reporting

Please select the one below that is the best fit for your research. If you are not sure, read the appropriate sections before making your selection.

☒ Life sciences ☐ Behavioural & social sciences ☐ Ecological, evolutionary & environmental sciences

For a reference copy of the document with all sections, see nature.com/documents/nr-reporting-summary-flat.pdf

Life sciences study design

All studies must disclose on these points even when the disclosure is negative.

Sample size	We used genome sequencing data from UK Biobank and All of Us, which were the largest WGS cohorts available to us and provided ample statistical power to identify genetic modifiers of somatic repeat expansion. Our GWAS on somatic expansion of TCF4 included 40,231 UK Biobank participants and 8,217 All of Us participants. Sample sizes for instability analyses of other STRs are reported in Supplementary Data 5. Phenotypic associations of long repeat expansions were computed in a set of 421,364 unrelated individuals of EUR genetic ancestry in UK Biobank.
Data exclusions	We excluded individuals who had withdrawn at the time of our study. We restricted our GWAS on somatic expansion of TCF4 to long-allele carriers and those with EUR genetic ancestry; within All of Us, we additionally restricted to an unrelated set of individuals and required individuals have age >= 40.
Replication	We analyzed the All of Us data set to replicate the key (K76 and CKD) GLS associations identified in UK Biobank; both the K76 and CKD associations replicated.
Randomization	Not applicable to our study; participants were analyzed together and not allocated into groups.
Blinding	Not applicable to our study; all data were previously collected, and participants were not allocated into groups.

Reporting for specific materials, systems and methods

We require information from authors about some types of materials, experimental systems and methods used in many studies. Here, indicate whether each material, system or method listed is relevant to your study. If you are not sure if a list item applies to your research, read the appropriate section before selecting a response.

Materials & experimental systems

n/a	Involved in the study
☒	☐ Antibodies
☒	☐ Eukaryotic cell lines
☒	☐ Palaeontology and archaeology
☒	☐ Animals and other organisms
☒	☐ Clinical data
☒	☐ Dual use research of concern
☒	☐ Plants

Methods

n/a	Involved in the study
☒	☐ ChIP-seq
☒	☐ Flow cytometry
☒	☐ MRI-based neuroimaging

Plants

Seed stocks

Report on the source of all seed stocks or other plant material used. If applicable, state the seed stock centre and catalogue number. If plant specimens were collected from the field, describe the collection location, date and sampling procedures.

Novel plant genotypes

Describe the methods by which all novel plant genotypes were produced. This includes those generated by transgenic approaches, gene editing, chemical/radiation-based mutagenesis and hybridization. For transgenic lines, describe the transformation method, the number of independent lines analyzed and the generation upon which experiments were performed. For gene-edited lines, describe the editor used, the endogenous sequence targeted for editing, the targeting guide RNA sequence (if applicable) and how the editor was applied.

Authentication

Describe any authentication procedures for each seed stock used or novel genotype generated. Describe any experiments used to assess the effect of a mutation and, where applicable, how potential secondary effects (e.g. second site T-DNA insertions, mosiacism, off-target gene editing) were examined.
